# Mutational landscape of atherosclerotic plaques reveals large clonal cell populations

**DOI:** 10.1172/jci.insight.188281

**Published:** 2025-04-08

**Authors:** Lasse Bach Steffensen, Stephanie Kavan, Pia Søndergaard Jensen, Matilde Kvist Pedersen, Steffen Møller Bøttger, Martin Jakob Larsen, Maja Dembic, Otto Bergman, Ljubica Matic, Ulf Hedin, Lars van Brakel Andersen, Jes Sanddal Lindholt, Kim Christian Houlind, Lars Peter Riber, Mads Thomassen, Lars Melholt Rasmussen

**Affiliations:** 1 Department of Molecular Medicine, University of Southern Denmark, Odense, Denmark.; 2 Centre for Individualized Medicine in Arterial Diseases (CIMA),; 3 Department of Clinical Biochemistry and Pharmacology, and; 4 Department of Clinical Genetics, Odense University Hospital, Odense, Denmark.; 5 Clinical Genome Center, Department of Clinical Research,; 6 Department of Clinical Research, and; 7 Department of Mathematics and Computer Science (IMADA), University of Southern Denmark, Odense, Denmark.; 8 Department of Molecular Medicine and Surgery, Karolinska Institute and Karolinska University Hospital, Stockholm, Sweden.; 9 Department of Cardiothoracic Surgery, Odense University Hospital, Odense, Denmark.; 10 Department of Regional Health Research, Lillebælt Hospital, Kolding, Denmark.

**Keywords:** Genetics, Vascular biology, Atherosclerosis, Clonal selection, Genetic variation

## Abstract

The notion of clonal cell populations in human atherosclerosis has been suggested but not demonstrated. Somatic mutations are used to define cellular clones in tumors. Here, we characterized the mutational landscape of human carotid plaques through whole-exome sequencing to explore the presence of clonal cell populations. Somatic mutations were identified in 12 of 13 investigated plaques, while no mutations were detected in 11 non-atherosclerotic arteries. Mutated clones often constituted over 10% of the sample cell population, with genes related to the contractile apparatus enriched for mutations. In carriers of clonal hematopoiesis of indeterminate potential (CHIP), hematopoietic clones had infiltrated the plaque tissue and constituted substantial fractions of the plaque cell population alongside locally expanded clones. Our findings establish somatic mutations as a common feature of human atherosclerosis and demonstrate the existence of mutated clones expanding locally, as well as CHIP clones invading from the circulation. While our data do not support plaque monoclonality, we observed a pattern suggesting the coexistence of multiple mutated clones of considerable size spanning different regions of plaques. Mutated clones are likely to be relevant to disease development, and somatic mutations will serve as a convenient tool to uncover novel pathological processes of atherosclerosis in future studies.

## Abstract 2

Deep whole-exome sequencing of human atherosclerotic plaques reveals that the expansion of large mutated clones within plaque tissue is an intrinsic feature of human atherosclerosis.

## Graphical abstract

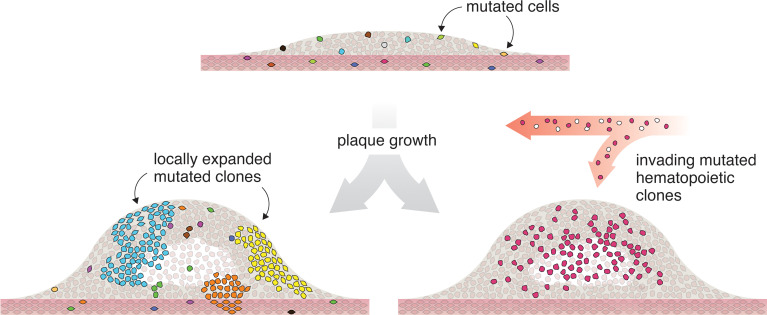

## Introduction

Atherosclerosis is the focal accumulation of lipids, fibrous tissue, and cells in the intimal layer of arteries. The disease develops over decades and may suddenly manifest as myocardial infarction or ischemic stroke, which are leading causes of death in the world (1).

The cellular component of atherosclerotic plaques originates from both circulating myeloid cells and local vascular cells, in particular smooth muscle cells (SMCs) recruited from the local medial layer (2). Recent advances in the field have revised our perception of both sources (3): First, through the use of multicolor lineage tracing in mouse models, it was unambiguously demonstrated that all SMC-derived plaque cells are progeny of few medial SMCs (4, 5), and that cell phenotypes within a clone could span both α-smooth muscle actin–positive (Acta2-positive) SMCs of the cap and Acta2-negative cells in the plaque interior. Although these observations were made in mouse models of atherosclerosis, they are compatible with the hypothesis of monoclonality in human atherosclerosis proposed by Benditt and Benditt 50 years ago (6). In their study, Benditt and Benditt leveraged X chromosome inactivation (X-inactivation) in women to uncover the dominance of 1 of the 2 isoforms of X-linked *G6PD* in bulk plaque tissue. This dominance was contrasted by the balanced expression of both isoforms in blood cells and non-atherosclerotic arteries.

Second, several lines of evidence now support an important role for clonal hematopoiesis of indeterminate potential (CHIP) in atherosclerotic cardiovascular disease (7–9). Individuals with CHIP carry expanded hematopoietic clones, often harboring mutations in epigenetic regulators (e.g., *DNMT3A* and *TET2*) (10), which, besides conferring a selective advantage, render myeloid cells more inflammatory and proatherogenic (11). These recent advances hint at the involvement of clonal cell populations in the pathology of atherosclerosis, but the presence and the extent of clonal populations participating in human atherosclerosis remain elusive.

Somatic mutations represent a change in the DNA sequence that occurs after conception. Somatic mutations can arise from various factors, including errors during DNA replication or exposure to environmental factors like radiation or chemicals (12). If not immediately corrected by the cellular repair machinery, somatic mutations become permanently integrated into the cell’s genome and are passed on to all its progeny, leading to potential changes in cell function and the risk of cancer development (13). The characterization of tumor mutational landscapes through DNA sequencing is the foundation for studying clonal and subclonal dynamics within tumors (14, 15), identifying cancer driver genes, and monitoring residual or recurrent disease, e.g., by circulating tumor DNA analysis. All these analyses rely on the undeniable premise that all cells harboring a given mutation share a common ancestral cell that originally acquired the mutation and that amplification of a mutation within an organism can only occur by cell proliferation.

Recent studies have revealed that aside from tumors, somatic mutations and clonal cell populations also exist in a variety of normal-appearing tissues such as blood (16) (CHIP), skin (17), prostate (18), bladder (19), and esophagus (20), and in various non-tumorous pathological conditions including chronic liver disease (21), inflammatory bowel disease (22), and endometriosis (23). Additionally, the burden of somatic mutations and the sizes of these clones have been observed to increase with age, suggesting a potential involvement in age-related declines in organ function (20, 24).

In this study, we hypothesized that, similar to bulk DNA sequencing of tumors, we could discern somatic mutated cellular clones constituting substantial portions of atherosclerotic plaque samples. Furthermore, we hypothesized that exploring the somatic mutational landscape of plaques might offer unprecedented insights into the potential role of clonal cell populations in atherosclerosis.

To investigate our hypothesis, we conducted deep whole-exome sequencing (WES) of bulk DNA extracted from human atherosclerotic plaques and non-atherosclerotic arterial tissue. Our initial focus was on plaque- and arterial tissue–confined mutations that were undetectable in buffy coat (blood leukocytes) DNA from the same patients. Subsequently, we identified CHIP carriers within the study cohort and investigated the contribution of hematopoietic clones to the plaque cell population.

## Results

### Somatic mutations and locally expanded clonal cell populations are inherent features of atherosclerosis.

**Figure 1:**
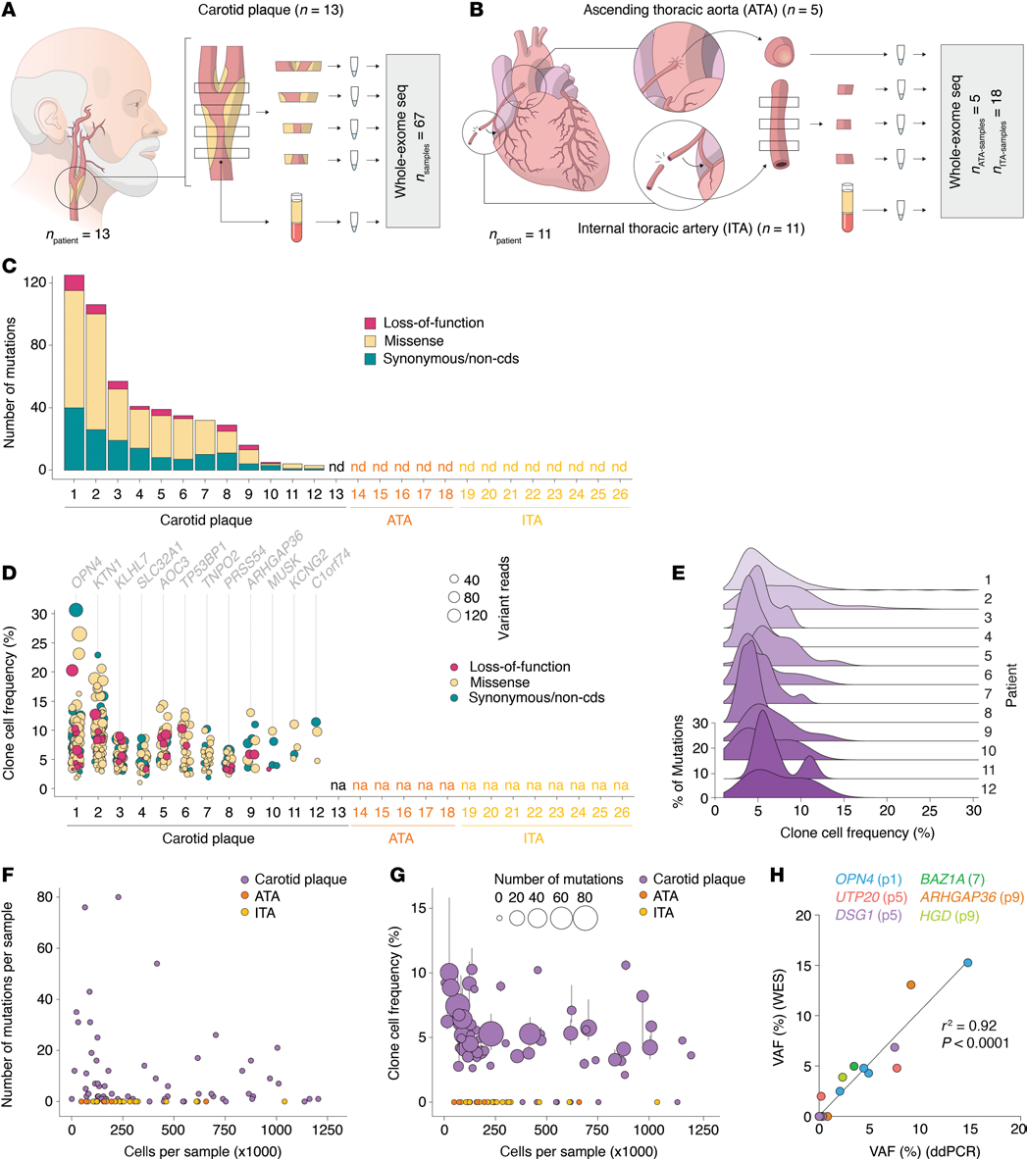
Somatic mutations and locally expanded clonal cell populations are inherent features of atherosclerosis. (**A**) Carotid atherosclerotic plaques from 13 patients undergoing carotid endarterectomy were segmented into 1–4 samples. DNA extracted from these segments was analyzed by whole-exome sequencing, with patient-matched buffy coat DNA serving as reference. (**B**) Non-atherosclerotic arterial tissue samples were obtained from ascending thoracic aortas (ATAs, *n* = 5) and internal thoracic arteries (ITAs, *n* = 6) of 11 patients undergoing coronary artery bypass surgery. ITA samples were subdivided into an average of 3 samples each, leading to a total of 18 ITA samples. (**C**) Bar plot showing the number of plaque- or arterial tissue–confined somatic mutations (i.e., mutations not identified in patient-matched buffy coats) for each patient. (**D**) Frequency of clonal cells carrying a specific somatic mutation in plaque samples for each patient. Mutation effect is indicated by dot color, and variant reads is indicated by dot size. For each patient, the mutated gene representing the highest clonal cell frequency is shown. (**E**) Density plot showing the distribution of clonal cell frequencies for each patient in which mutations were detected. (**F**) The number of mutations detected per sample is plotted against the estimated number of sample cells, which was calculated based on the sample’s DNA yield and assumes an average of 6.6 pg DNA per cell. (**G**) The median clonal cell frequency of mutations detected in each sample plotted against the estimated number of sample cells. Vertical bars indicate the interquartile range, while dot sizes show the number of mutations per sample. (**H**) Correlation between VAFs obtained from ddPCR and VAFs obtained from whole-exome sequencing of the same plaque samples was analyzed for 6 specific mutations by linear regression analysis. Extended analyses are provided in Supplemental Figure 2.

WES was performed on DNA extracted from carotid plaques obtained from 13 patients undergoing carotid endarterectomy (Figure 1A) and non-atherosclerotic ascending thoracic aortas (ATAs) (*n* = 5) and internal thoracic arteries (ITAs) (*n* = 6) obtained from 11 patients undergoing coronary artery bypass surgery (Figure 1B) (patient characteristics are provided in Supplemental Table 2, and histology of plaque specimens is provided in Supplemental Figure 1; supplemental material available online with this article; https://doi.org/10.1172/jci.insight.188281DS1).

At tissue harvest, arterial tissues were longitudinally segmented into 2- to 5-mm sections, with alternate segments either frozen or processed for histology. As a result, the frozen segments (which were used for WES in this study) were spaced 4–10 mm apart within the intact plaque.

Upon inspection of the frozen plaque segments, 4 displayed sufficiently well-preserved morphology, allowing for further dissection under the microscope while reliably maintaining their morphological context. The remaining segments, where plaque layers could not be readily separated, were either sequenced as intact segments or, if too large, divided into more pieces to facilitate DNA purification and sequenced independently. These segments represented bulk plaque tissue, encompassing all layers, and allowed us to study the distribution of putative somatic mutations along the length of the specimens.

In total, the number of independently sequenced samples was 67 plaque-derived samples, 5 ATA samples, and 23 ITA samples. DNA extracted from the buffy coat (blood leukocytes) of each patient was used as a reference for the analysis of sequencing data. We identified somatic mutations in 12 of the 13 carotid plaques investigated and, on average, 37.8 (range: 0–125, median: 33.5, IQR: 13.25–45) somatic mutations per plaque; however, no mutations were detected in any of the non-atherosclerotic ATA and ITA tissue samples (Figure 1C). Identified mutations were primarily point mutations (96%), but also insertions/deletions of various lengths (Supplemental Table 3). Clonal cell frequencies, calculated from variant allele frequencies (VAFs) of identified mutations, ranged from 1% to 30.6% (median: 5.4%, IQR: 4%–7.8%) (Figure 1, D and E, and Supplemental Table 3). As expected, the number of detected mutations and clonal cell frequencies were inversely correlated with the estimated number of sample cells (as calculated assuming 6.6 pg DNA per cell) (Figure 1, F and G). Six mutations displaying variable VAFs across samples within a patient were chosen for validation by droplet digital PCR (ddPCR), and the VAFs closely mirrored those obtained from the WES analysis (Figure 1H and Supplemental Figure 2). No correlation was found between age, sex, clinical characteristics, or cause of death, and the number of somatic mutations, likely attributable to the limited size of the study cohort.

### Mutated clones span several regions of the plaque.

**Figure 2:**
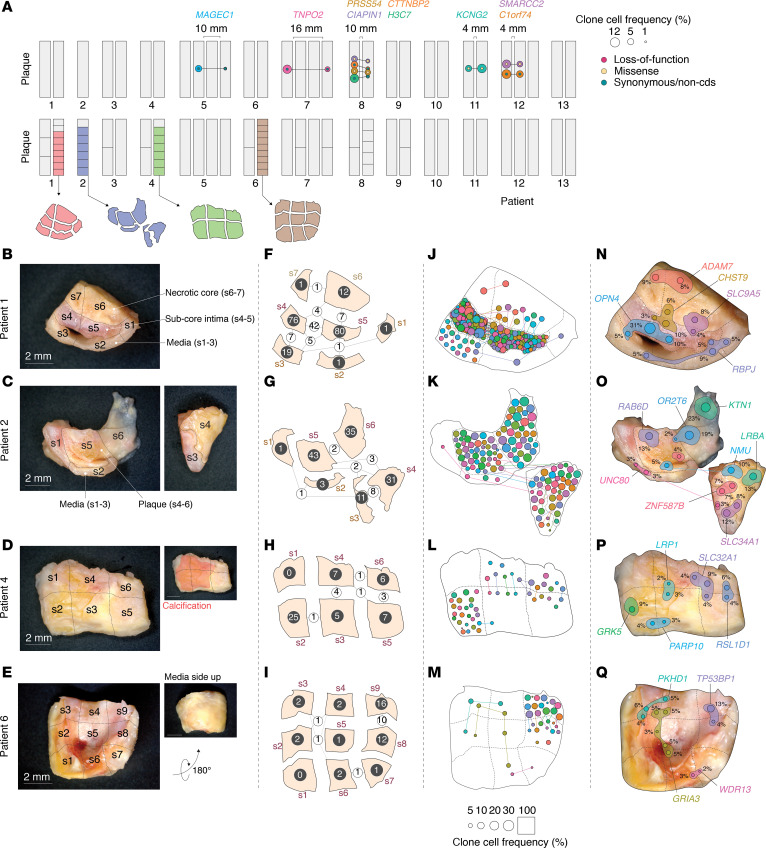
Mutated clones span several regions of the plaque. (**A**) In 5 patients, somatic mutations were detected in multiple plaque segments from the same individual. In the top panel, mutations shared across more than one segment are represented as interconnected colored dots. The colors of the dots correspond to the associated gene symbols, and the size of the dots reflects the frequency of the clonal cells. The mutation effect is indicated by smaller dots within the main mutation dots. Intersegment distances are shown, providing an estimate of the physical extent of clonal populations. The bottom panel illustrates how each segment was subdivided into the samples analyzed by whole-exome sequencing. One plaque segment from patients 1, 2, 4, and 6 exhibited sufficient morphological integrity, allowing for dissection while maintaining the morphological context (colored). (**B**–**E**) Depicted are images of the plaque segments, illustrating the division process into distinct samples. (**F**–**I**) The number of mutations identified in each sample is represented by white numbers, while the number of shared mutations between samples is denoted by black numbers and the thickness of the intersample connection lines. (**J**–**M**) Mutations detected in each sample are shown as dots, with specific mutations shared among samples connected by lines. The size of each dot corresponds to the clonal cell fraction it represents, as indicated in the key. Of note, the sample area does not correspond to 100%, as it was increased to fit the dots. (**N**–**Q**) Possible interpretations of the distribution of clones harboring selected mutations are depicted. The dot color and size mirror the key in **J**–**M**. Additionally, a shaded area has been added for which the size corresponds to the clonal cell frequency for each sample, setting the sample area to 100%. The genes that are mutated are indicated.

For several plaques, specific mutations were identified in more than one segment. For some of the clones harboring these mutations, this would require a physical clonal extent of at least 4–16 mm (Figure 2A).

Four plaque segments from different patients (patients 1, 2, 4, and 6) (Figure 2A) maintained adequate morphological integrity to allow for a detailed dissection into subsamples. This approach preserved the morphological context of each resulting sample (Figure 2, B–E). The distribution of mutations across these samples provided the following morphological insights:

### Patients 1 and 2.

Samples from patients 1 and 2 exhibited distinct layering when observed macroscopically. Consequently, it was possible to separate the medial layer from the overlying plaque tissue, which could be further subdivided (Figure 2, B and C). In both patients, the medial layer samples exhibited markedly fewer mutations compared with the overlying plaque samples (Figure 2, F, G, J, and K).

In the plaque segment of patient 1, the medial layer was divided into 3 samples (s1–s3), and the plaque was divided into 2 subcore intimal samples (s4 and s5) and 2 necrotic core samples (s6 and s7) (Figure 2B). Two samples from the medial layer (s1 and s2) had only 1 mutation (*RBPJ^mut^*), which extended throughout all 3 media samples (s1–s3) and protruded into intima s5 (Figure 2, F and J; a possible interpretation of the *RBPJ^mut^* clone extension is depicted in Figure 2N). The third sample from the media (s3) had 19 mutations, 12 of which were also present in the overlying intima sample (s4) (Figure 2F). A subset of these was also present in the neighboring intima sample (s5). In particular, a mutation in *OPN4* was present in 5% of cells in s3, 31% of cells in s4, and 10% of cells in s5, suggesting the presence of a large media-intima–spanning clone (possible interpretation of the *OPN4^mut^* clone is illustrated in Figure 2N).

The 2 subcore intima samples (s4 and s5) exhibited a high abundance of somatic mutations, and 51 mutations overlapped between these 2 samples (Figure 2, F and J). From the subcore intima, 11 mutations extended into the overlying necrotic core (exemplified by *CHST9^mut^* and *SLC9A5^mut^*, with a possible interpretation of mutation-carrying clones is illustrated in Figure 2N). In contrast to the subcore region, we only detected a single independent (i.e., not found in other regions) mutation (in *ADAM7*) in the necrotic core, which spanned both necrotic core samples (s6 and s7) (Figure 2, F and J; possible interpretation of the *ADAM7^mut^* clone is illustrated in Figure 2N).

Similar to patient 1, the media samples (s1–s3) of patient 2 (Figure 2C) had relatively few mutations (Figure 2, G and K). A single mutation (in *UNC80*) spanned all media samples but was not found in any plaque samples (Figure 2, G and K). Media s3 had 10 mutations in common with plaque s4. The most abundant of these mutations was *ZNF587B^mut^* (which also extended into s5) and *SLC34A1^mut^* (possible interpretation of clones carrying these mutations is illustrated in Figure 2O). In contrast to the medial layer, numerous mutations were identified in the 3 plaque samples (s4–s6), with modest intersample overlap (Figure 2, G and K). However, several plaque mutations were of considerable size, e.g., *KTN1^mut^* (s5), *OR2T6^mut^* (spanning s5 and s6), *RAB6D^mut^* (s6), and *NMU^mut^* (spanning s4 and s5), with clonal cell frequencies of 23%, 19%, 13%, and 10%, respectively (possible interpretation of clones carrying these mutations is illustrated in Figure 2O).

### Patients 4 and 6.

Plaque segments from patients 4 and 6 were sectioned using a distinct strategy. They were cut into approximately equal-sized samples, ensuring that each piece encompassed all vessel layers (media and overlying plaque) uniformly (Figure 2, D and E).

In general, fewer mutations were detected in the plaque segments from these 2 patients. The largest clone detected in patient 4 was a *GRK5^mut^*-carrying clone constituting 9% of cells in s2 (Figure 2, H and L). Other mutations overlapped between neighboring regions, e.g., *PARP10^mut^* (s2 and s3), *LRP1^mut^* (spanning s3 and s4), *SLC32A1^mut^* (spanning s4–s6), and *RSL1D1^mut^* (s5 and s6) (possible interpretation of clones carrying these mutations is illustrated in Figure 2P).

In patient 6, we observed a mutation in *GRIA3*, which spanned 5 samples (s2–s6). In this plaque, mutations were more confined to s8 and s9, which had 10 overlapping mutations. Examples of other sample-overlapping mutations of patient 6 are *PKHD1^mut^* (s2–s4), *WDR13^mut^* (s6 and s7), and *TP53BP1^mut^* (s8 and s9) (possible interpretation of clones carrying these mutations is illustrated in Figure 2Q).

For several samples, the sum of clonal cell frequencies calculated from VAFs of each mutation exceeded 100%. Therefore, at least some clones must carry more than one mutation. While the bulk sequencing analysis presented here does not allow for a direct assessment of the mutational architecture of different clones, we observed distinct patterns shared by groups of mutations, suggesting that certain mutations may be carried within the same clone.

In particular, mutations identified in the same samples and exhibiting similar magnitude and intersample differences in clonal cell frequencies (Supplemental Figure 3, A–D) are likely carried by the same clone, as proposed in Supplemental Figure 3, E–H. As an example, it is reasonable to assume that the clone carrying *OPN4^mut^* in patient 1 also carries *POU4F3^mut^*, *ZNF800^mut^*, and *LRP1^mut^*, since these mutations are present in the same samples and show comparable differences in clonal cell frequency between samples (Supplemental Figure 3A).

### Non-random distribution of genes mutated in plaque tissue.

After establishing the existence of locally expanded mutated clones, we sought to explore whether somatic mutations were clone drivers, i.e., conferring a selective advantage (analogous to neoplasms), or merely amplified by coincidence as passengers in a clone expanding by other mechanisms.

We did not detect any mutational hotspots (as in CHIP) (10) and certain types of cancer (25). However, 21 of the mutated genes appeared in the Integrative Onco Genomics database of 619 mutational cancer driver genes (25) (χ*^2^* test: *P* = 0.015), and 32% of the genes have previously been linked to a Mendelian disorder (Supplemental Table 4).

**Figure 3:**
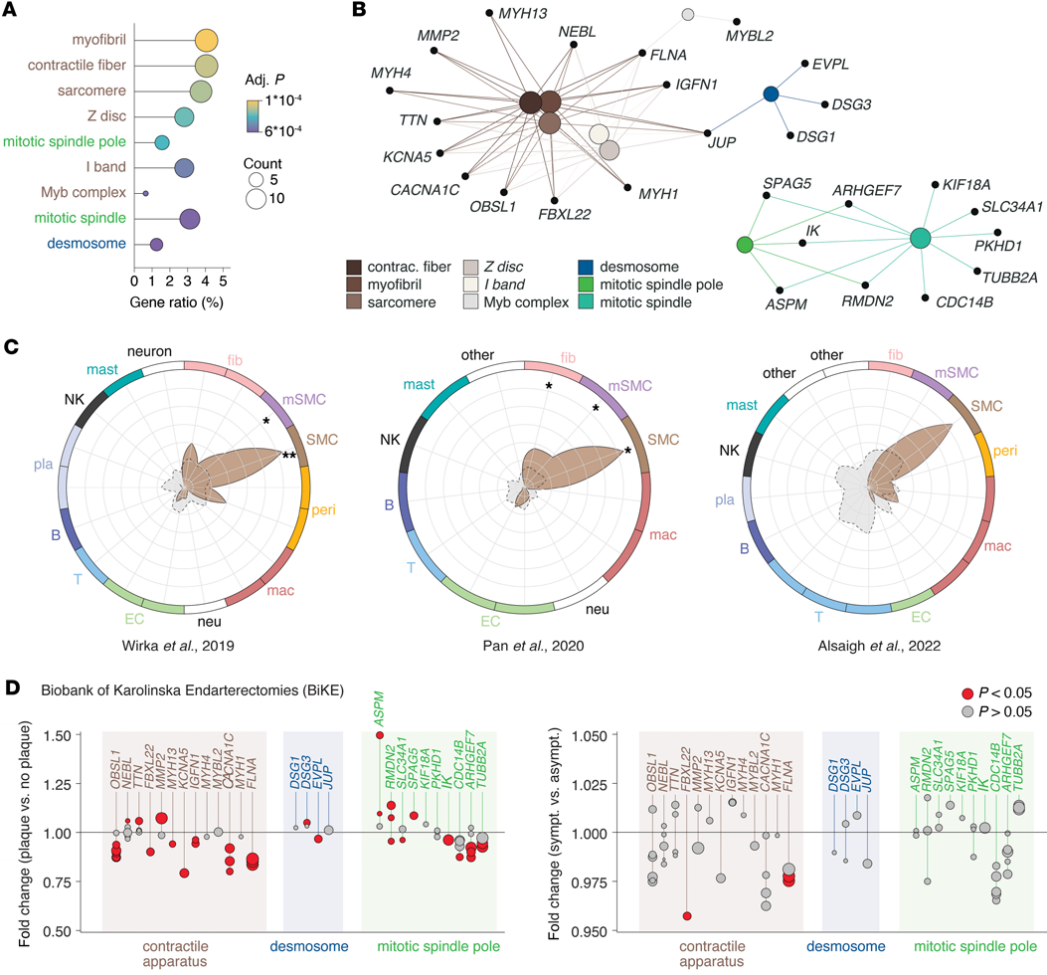
Non-random distribution of genes mutated in plaque tissue. (**A**) Dot plot showing the enriched gene ontology terms of the 334 genes having a loss-of-function or missense mutation across all patients. Dot sizes indicate number of mutated genes for each term. The ratio shows the coverage of a given term by mutated genes, and dot colors indicate significance level of the enrichment (Kolmogorov-Smirnov–like running sum statistic using Benjamini-Hochberg to control for false discovery rate). (**B**) Network plot showing enriched gene ontology terms and the mutated genes belonging to each term. (**C**) To evaluate the expression pattern of mutated genes, 3 single-cell RNA sequencing datasets of human plaques were used. The mean expression level of genes belonging to the contractile apparatus, in which we found a mutation, is plotted for each cell population. The mean expression level of all genes of the atherosclerosis transcriptome was plotted for each dataset in gray for comparison. Asterisks indicate that contractile genes have significantly higher expression as compared with the background gene population. *P < 0.05, **P < 0.01 by Mann-Whitney *U* test. fib, fibroblast; mSMC, modulated SMC; peri, pericyte; mac, macrophage; neu, neutrophil; EC, endothelial cell, T, T cell; B, B cell; pla, plasma cell; NK, natural killer cell; mast, mast cell; other, non-annotated clusters in original publications. (**D**) Differences in the expression of genes associated with the 3 enriched ontology terms (contractile apparatus, desmosome, and mitotic spindle pole) were analyzed between carotid plaques and non-lesioned control arteries (left) and between carotid plaques from symptomatic and asymptomatic patients (right). Expression levels for individual microarray probes are displayed for each gene. Red data points indicate significant differences between conditions (Student’s *t* test), while the size of the data points reflects the mean expression level for each probe across all plaque samples.

Over-representation analysis of all 334 genes identified across the 13 patients having either a missense or a loss-of-function mutation resulted in significant enrichment of terms associated with the contractile apparatus, mitotic spindle pole, and desmosome (Figure 3, A and B). To investigate which cell populations in atherosclerotic lesions express the genes driving these enrichments, we leveraged 3 single-cell RNA sequencing datasets of human plaques (26–28).

The expression of mutated genes associated with the contractile apparatus was markedly higher in SMCs and modulated SMCs in 2 datasets, with a similar trend in the third (Figure 3C), while mutated genes associated with desmosome (*n* = 4) and mitotic spindle pole (*n* = 10) were less consistent between datasets, without significantly higher expression in any cell population (Supplemental Figure 4). To further explore the potential involvement of these genes in atherosclerosis, we compared bulk transcriptomic expression of carotid plaque tissue (*n* = 127) with control, non-lesional arterial tissue (*n* = 10) and found several of the genes to be differentially expressed (Figure 3D). When comparing carotid plaques from symptomatic patients (*n* = 87) to those from asymptomatic patients (*n* = 40), fewer differences in gene expression were observed (Figure 3D); however, *FBXL22* and *FLNA* were significantly downregulated in symptomatic patients.

### Circulating CHIP clones contribute to the plaque cell population.

**Figure 4:**
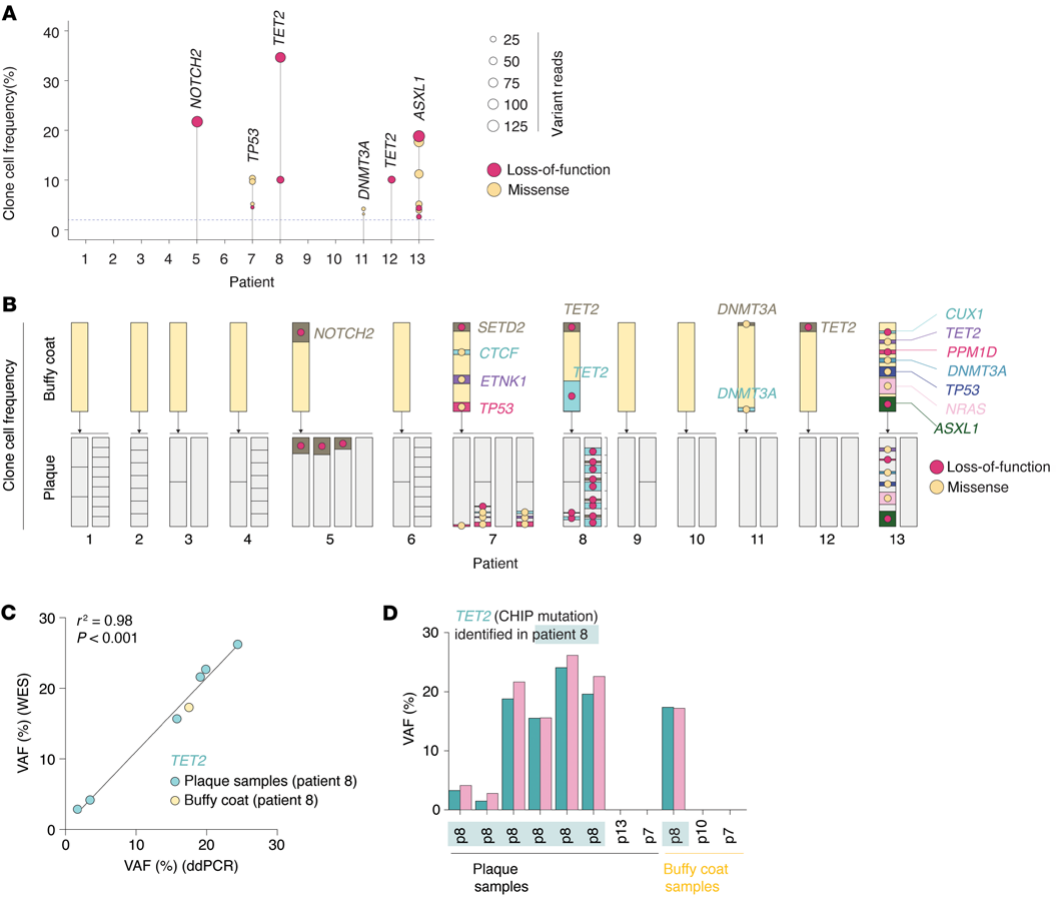
Contribution of clonal hematopoiesis of indeterminate potential (CHIP) clones to the carotid plaque cell population. (**A**) Six of the 13 plaque donors were CHIP carriers. The plot shows clonal cell frequencies of CHIP mutations detected in buffy coat (blood leukocytes) DNA. For each patient, the identity of the mutated gene with the highest clonal cell frequency is shown. Dot sizes indicate variant reads, and colors indicate the type of mutation. The dashed line shows the defined limit of detection. (**B**) CHIP clonal cell frequencies in buffy coats (yellow bars) and in subdivided plaque segments (gray bars). Clonal cell frequencies are represented by the proportion of colored area within yellow bars or gray subsamples (relative to the subsample bar area). Mutation type is indicated by dot color. (**C** and **D**) Validation of VAFs for a *TET2* loss-of-function mutation, identified in patient 8, was conducted by comparing VAFs derived from ddPCR with those from whole-exome sequencing of both buffy coat and plaque samples from patient 8 (by linear regression analysis), as well as from samples of other patients (serving as negative controls).

We next investigated the contribution of circulating CHIP mutation–carrying cells to the plaque cell population. By screening for mutations in 78 previously established CHIP genes (10) using buffy coat DNA, we found 6 of the 13 patients to be CHIP carriers, and several CHIP mutations were detected in each of these patients (Supplemental Table 5). Buffy coat–derived VAFs ranged from 1.3% to 17% (median: 3.7%, IQR: 2.2%–6.3%), corresponding to clonal cell frequencies of 2.6%–34% (median: 7.4%, IQR: 4.4%–12.5%) (Figure 4A). In several cases, we identified the same mutations in patient-matched plaque tissue samples. These observations suggest that circulating CHIP-mutated cells infiltrate atherosclerotic plaques, and in some cases, constitute a substantial portion of the plaque cell population. For example, in the case of patient 5, a *NOTCH2* loss-of-function mutation had a 21.6% buffy coat clonal cell frequency, and the same mutation was detected in 3 out of 4 plaque samples from the same patient, where it contributed to 13.4%–18.4% of the plaque sample cell population (Figure 4B). Validation of a selected *TET2* mutation by ddPCR confirmed VAF values from WES across all samples from the patient carrying this mutation (Figure 4, C and D). In contrast to the enrichment of plaque-confined mutations in genes that are highly expressed in vascular cells, the identified CHIP mutations were in genes that exhibited increased expression in macrophage populations, as demonstrated by 2 single-cell RNA sequencing datasets (Supplemental Figure 5).

## Discussion

In this study, we employed WES to DNA extracted from bulk human atherosclerotic tissue samples to reveal the prevalence of somatic mutations as a common feature of plaques. The study design facilitated the discovery of mutated cell populations not detectable in patient-matched buffy coats, indicating local clonal expansion of the mutated cell within the plaque tissue. By employing methodology from the cancer field, VAFs were used to determine the relative number of mutation-carrying cells in samples, and the physical spread of clones was estimated by detecting specific mutations in multiple samples from the same plaque. These findings not only place atherosclerosis among a growing list of non-cancerous diseases where somatic mutations may play a role (29), but also shed light on processes that contribute to the cellular composition of human plaques, i.e., asymmetric cellular proliferation leading to large portions of plaque cells having a shared ancestral cell, that at some point acquired a mutation.

Our findings are predicated on the assumption that all cells harboring a given mutation originate from a common ancestral cell. While identical mutations can occur across different patients with the same cancer type, it is improbable that the same mutation would independently arise multiple times within a single lesion. This is further supported by our observation that no single mutation was found to recur across different patients within our cohort.

The occurrence of various types of DNA damage in atherosclerosis has previously been reported. These include single- and double-stranded breaks, copy number variations, and base modifications, with the oxidative environment proposed as a key mutagenic cause (30). Shah et al. demonstrated that reactive oxygen species not only damage DNA but also impair DNA repair mechanisms in SMCs (31). DNA damage may in general exacerbate atherosclerosis by increasing inflammation (30), but it may also affect the disease by inducing tumor-like characteristics in certain cell types, such as SMCs (32). For instance, experimental studies have demonstrated that inducing the oncogenic *Kras^G12D^* mutation promotes SMC proliferation within plaques, contributing to disease progression in mouse models (32). The present study provides direct evidence of the plaque-confined expansion of cells carrying specific mutations through proliferation resulting in large mutated clones in human atherosclerotic plaques.

Another significant finding from our study concerns the presence of CHIP-mutated hematopoietic clones in atherosclerotic plaque tissue. The prevailing understanding is that individuals with CHIP mutations face an increased risk of atherosclerotic cardiovascular disease due to increased leukocyte proinflammatory activity, exacerbating plaque formation (33). This hypothesis is supported by direct evidence of CHIP-carrying cells being present in human atherosclerotic plaques, as we demonstrate here and as recently reported by Dederichs et al. (34). Among the mutations described as plaque-confined identified in this study, 2 mutated genes (*BCOR* and *U2AF1*) are known CHIP genes. While these mutations may have originated within the plaque tissue itself, it is also possible that they were present in circulating cells at undetectable levels and subsequently expanded locally through proliferation within the plaque tissue.

Although our study relied on bulk plaque samples, we occasionally had the opportunity to subsample the medial layer and distinct areas of the plaque. Despite the limited sample size used for this morphology-coupled analysis, we observed that mutations were concentrated in the (subcore) intimal layer, as opposed to the necrotic core, while the underlying medial layer exhibited relatively few mutations. Importantly, however, this observation was restricted to patients 1 and 2 (Figure 2, F, G, J, and K), as comparisons between these compartments were only feasible for these donors. Interestingly, the observed pattern of certain mutations, such as the *OPN4* mutation (Figure 2N), which spanned the media and subcore intima samples, is similar to that of SMC growth/migration, and aligns with the known capacity SMCs to proliferate and form clones in experimental atherosclerosis (4, 5). However, further investigations are imperative to conclusively pinpoint the mutated cell types responsible for these observed mutation patterns. Interestingly, the observation that the medial layer samples exhibited few mutations compared with the overlying plaque supports the validity of the numerous mutations identified in the adjacent plaque tissue and supports the hypothesis that clonal expansion is related to plaque formation rather than the alternative explanation of large preexisting mutated clones in the predisease artery. We consider potential cross-sample DNA contamination in the overall tissue handling to be negligible and have no effect on conclusions regarding clonal spread, since mutations were always only occurring in neighboring samples, and at a relatively high VAF, while other samples from the same plaque were negative.

Interestingly, some plaques exhibited bimodal distribution in clonal cell frequency (Figure 1E), as seen in patients 3, 7, and 11. This observation could indicate the coexistence of small and large clones within plaques, as possibility supported by the intraplaque mapping of clones shown in Figure 2. Alternatively, it could reflect subclone formation, where new mutations arise at later stages of clonal expansion, resulting in lower VAFs compared with mutations acquired earlier in the expansion process.

The mutational landscape observed in carotid plaques stands in stark contrast to our analyses of non-atherosclerotic ATAs and ITAs, where no mutations were detected despite that sample sizes (as determined by DNA yields) and sequencing depths were comparable across all samples investigated. It is important to note, however, that we used a VAF threshold of 1% as the cutoff for mutation detection in our analyses. Therefore, it remains plausible that non-atherosclerotic arteries (and atherosclerotic samples) harbor small, mutated clones, which remain undetected in our analysis. Indeed, many other non-diseased tissues have been reported to contain clonal populations of a few hundred cells (16). However, this does not undermine our conclusion that several plaque segments exhibit over 10% of cells carrying specific mutations and must, therefore, have a shared ancestral cell that initially acquired the mutation. It is also important to note that our analyses of ATAs and ITAs may not accurately represent the mutational profile of more atherosclerosis-prone sites, such as the carotid arteries. Furthermore, it is important to take into account that the donors of ATAs and ITAs had a mean age of 67.1 years, whereas plaque donors were significantly older, with a mean age of 75.8 years. Mutational burden is known to increase with age in other tissues; however, the modest difference in mean age and the significant overlap — over 25% of individuals in each group are between 68 and 72 years old — make it unlikely that the observed absence of mutations in non-atherosclerotic tissues, contrasted with numerous mutations and high VAFs in 12 out of 13 plaque samples, can be attributed to age differences between the 2 donor groups.

The variability in the number of mutations observed between plaques, with one plaque exhibiting no mutations at all, suggests that mutations may not be essential for plaque development. While atherosclerosis is primarily driven by lipid accumulation and inflammation in its early stages, somatic mutations could potentially accelerate plaque development. This is analogous to how conditions like diabetes and smoking, although not essential for atherosclerosis to form, can accelerate its progression. However, this hypothesis necessitates further experimental investigation to establish definitive causative relationships.

The identification of somatic mutations using next-generation sequencing methods can sometimes lead to false discoveries. To address this concern, we adopted stringent criteria for variant calling and only included mutations that were validated through independent rounds of sequencing. Additionally, the frequent observation of the same mutation appearing in several neighboring plaque samples, while never detectable in samples from other patients, argues for a robust mutation detection design. Moreover, the significant disparity in the number of identified mutations among patients, coupled with the absence of mutations in certain samples, indicates a minimal false-positive rate. Furthermore, we validated 7 mutations using ddPCR, consistently obtaining VAF values that closely matched those obtained from WES.

Our findings pose a critical question regarding whether mutations act as drivers of clonal expansion, providing a selective advantage, or as passengers accompanying clones expanding through other mechanisms. While we did not identify hotspot clone driver genes, meaning the same gene mutated in several plaques, our analyses revealed a non-random distribution of mutations across the exome: First, among the nearly 492 genes where mutations were found, 21 are known cancer driver genes. Second, we identified the enrichment of terms related to the contractile apparatus (with genes underlying the enrichment being significantly more highly expressed in SMCs, modulated SMC populations, and fibroblasts as compared with the background transcriptome), desmosomes, and mitotic spindle poles among missense or loss-of-function mutations. Analysis of bulk transcriptomic data from carotid plaques and control, non-lesional arteries revealed a reduced expression of several contractile genes in plaques. This reduction is likely attributable to a combination of inflammatory cell infiltration, diluting the SMC-specific gene expression, and the well-established downregulation of contractile apparatus genes by SMCs that have undergone phenotypic modulation. The downregulation of several genes associated with the contractile apparatus, in which we identified mutations, suggests that these genes may influence SMC behavior in atherosclerosis. Consequently, it is plausible that missense mutations in these genes could exacerbate SMC-driven processes, thereby contributing to plaque progression. Disruption and misfolding of components of the contractile apparatus have been linked to atherosclerosis-promoting SMC phenotypes (35). Notably, only a few of the genes were differentially expressed between symptomatic and asymptomatic plaques. While this may be due to high variability and, consequently, limited statistical power, it could imply that somatic mutations play a role in plaque growth but have less impact on plaque vulnerability.

Although the pattern of regulation is less consistent with genes associated with desmosomes and mitotic spindle poles, these genes also appear to be differentially expressed in plaques as compared with non-lesional arteries. Disruption of cell adhesion is crucial in malignant transformation and cancer progression (36), and the same mechanisms could be at play in atherosclerosis. This process allows cells to detach from their natural microenvironment, losing signals that maintain quiescence. Additionally, mutations in genes regulating the formation of the mitotic spindle pole can have significant consequences, potentially leading to genomic instability or disrupted mitotic regulation (34). It is important to note that while the genes associated with the contractile apparatus are significantly highly expressed in fibroblasts, SMCs, and modulated SMCs, this does not imply that mutations in these genes would not disrupt the function of other cell types. Taken together, our results show that somatic mutations are not randomly distributed throughout the exome. However, determining whether somatic mutations play a causal role in clonal expansion necessitates further investigation. Genomic regions of open chromatin have been proposed to be more susceptible to DNA damage (37). Conversely, it has also been suggested that open chromatin regions benefit from enhanced access to repair mechanisms (38), adding complexity to this hypothesis. We cannot rule out the possibility that the observed enrichment, such as in genes highly expressed in SMCs, is driven by increased mutagenesis associated with open chromatin in these regions.

The hypothesis of monoclonality in atherosclerosis was initially proposed by Benditt and Benditt over 50 years ago (6). All studies aiming to explore the hypothesis using human tissue, including the original study by Benditt and Benditt, have leveraged common variants in genes located on the X chromosome and investigated X-inactivation in plaques from females heterozygous for the variant. Originally, the electrophoretically separable isoforms of glucose-6-phosphate dehydrogenase encoded by the X-linked *G6PD* gene were utilized to demonstrate that most bulk plaque samples exhibited only one isoform, contrasting with balanced expression of both isoforms in non-atherosclerotic arterial tissue and blood samples (6, 39), suggesting monoclonality of plaque cells. However, this was later contested by Thomas et al., who found both isoenzymes in plaque samples (40). Murry et al. relied on a tandem repeat variant within the androgen receptor gene (*AR*), yielding differential PCR band sizes (41). Only one allele was amplifiable per cell since X-inactivation involved heavy methylation and protection of the inactivated allele from restriction digest prior to PCR. In line with observations by Benditt and Benditt, and Pearson et al., they found medial samples to exhibit balanced X-inactivation, whereas most plaque samples showed expression of only one allele. Recently, Kawai et al. employed probe-based histological staining that could differentiate a small deletion in the X-linked biglycan gene (*BGN*) from the wild-type allele at the mRNA level (42). Although this study did not support the monoclonality of *BGN*-expressing cells (primarily SMCs) in atherosclerosis, they reported increased clonality in plaque areas compared with the medial layer. While our data do not support purely monoclonal plaques, we observed a pattern suggesting the coexistence of multiple mutated clones of considerable size spanning different regions of plaques.

Regardless of somatic mutations being causally involved in clonal expansion, our finding that they are abundantly present in most investigated plaques offers what we believe is a novel and highly convenient tool that can be harnessed to obtain previously unprecedented conclusions of cellular dynamics in atherosclerosis. Using somatic mutations as genetic tags overcomes all shortcomings of X-inactivation studies, which, although informative, do not reach the same level of conclusiveness as studies conducted in multicolor lineage tracing models in mice:

First, all cells carrying the same specific mutation share a common ancestral cell and are part of the same clone, unlike X-inactivation, where cells with the same allele inactivated may not be part of the same clone. Second, all X-linked analyses, regardless of being based on bulk samples or sections, rely on physical coherence of clonal cells to define them as clones. This can result in overestimating clone size when clones with the same X-inactivation intermix, and it can lead to underestimation or the inability to detect clones entirely when clones with different alleles inactivated intermix by migratory processes, or when non-clonal cells mix with clonal cells. However, neither of these scenarios impacts the ability to assess the size of a mutated clone through probing a somatic mutation. Third, if the plasticity of clonal cells is similar to that documented in mice (5), it is critical that the clone label remains measurable even if clonal cells adopt different cellular phenotypes. For instance, in the study by Kawai et al. (42), if a fraction of clonal SMCs phenotypically modulate and cease to express *BGN*, the clonal cells can no longer be detected. Fourth, while somatic mutations are widespread plaques (12 of 13 plaques investigated in this study), X-inactivation studies are limited to females heterozygous for the probed gene. Finally, DNA is a highly stable molecule, unlike RNA which can degrade. As mentioned by Kawai et al. (42), RNA degradation could affect the accuracy of their analyses.

In contrast, our investigation has unveiled the promising prospect of leveraging somatic mutations as genetic markers to directly achieve information about clonal evolution and cellular dynamics in atherosclerosis. Technologies that link somatic mutations in genomic DNA at the single-cell level with single-cell transcriptomics will be crucial for unlocking the full potential of using somatic mutations to understand the cellular dynamics of human atherosclerosis.

In recent decades, the epidemiology of atherosclerotic cardiovascular disease has shifted from primarily affecting middle-aged smoking, hypercholesterolemic, and hypertensive men to increasingly impacting older people (43). This transition is attributed to the success of lipid- and blood pressure–lowering medication, lifestyle changes, and to the aging population in most countries (43). Consequently, the residual cardiovascular risk is increasingly associated with age-related factors. The identification of CHIP as an age-associated risk factor for atherosclerotic cardiovascular disease underscores the significance of mutated clonal populations in the disease (7, 8). Our study demonstrates that mutated clones, besides invading from the circulation, also develop within the plaque tissue. However, the extent to which these processes contribute to disease development and residual risk warrants further investigation.

### Conclusions

This study reveals a previously uncharacterized aspect of human atherosclerotic pathology: the presence of plaque-confined somatic mutations and locally expanded mutated clones comprising significant portions of the plaque cell population. These insights shed light on previously overlooked facets of atherosclerosis, which could represent hitherto unknown targetable disease mechanisms. Furthermore, we highlight that regardless of their role, the mere existence of somatic mutations in human plaques can serve as highly warranted genetic tags, enabling the study of cellular dynamics in human atherosclerosis and providing unprecedented pathological insights.

## Methods

### Sex as a biological variable.

Our study examined male and female tissue donors, and similar findings are reported for both sexes.

### Patient sample processing.

Carotid plaque tissue and buffy coats from 13 later deceased patients who had undergone carotid endarterectomy either at the Department of Cardiac, Thoracic and Vascular Surgery, Odense University Hospital (OUH), or at the Department of Vascular Surgery, Kolding Hospital, were included in the study. The endarterectomies were performed under general anesthesia using the classic endarterectomy technique. This involved making a longitudinal incision, followed by instrumental dissection to remove the intima and approximately half of the media. The procedure was completed by suturing the distal edge of the intima and closing the incision with patch angioplasty.

Patients were admitted for surgery because of recent carotid plaque–associated symptoms (stroke, transient ischemic attack, or amaurosis fugax). No patients had a cancer diagnosis prior to the endarterectomy. Causes of death were postoperative complications (*n* = 3), cancer (*n* = 2; bladder and bile duct), cerebral infarction (*n* = 2), lung disease (*n* = 2; infection and COPD), chronic pancreatitis (*n* = 1), myocardial infarction (*n* = 1), and suicide (*n* = 1).

Immediately after surgery, the entire removed material was placed and kept in ice-cold Hanks Buffered Salt Solution containing 10 mM HEPES (Biological Industries, SKU BI-02-016-1A and BI-03-025-1B, respectively). Within 24 hours, the removed tissue was divided into 2-mm segments (patients 5, 7, 11, 12, and 13) or 5-mm thick segments (patients 1, 2, 3, 4, 6, 8, 9, and 10) using a custom-made device. Every third segment was frozen at –80°C and stored in the Odense Artery Biobank infrastructure of the Centre for Individualized Medicine in Arterial Diseases (CIMA) at OUH. Four plaque segments were further subdivided to assess the distribution of mutations while preserving morphological context. As for all tissue processing, this was conducted on clean, disposable surfaces using disposable scalpels under the microscope.

Blood was collected in EDTA vials, centrifuged at 4,000*g* for 10 minutes at 4°C, and buffy coats were collected using a pipette from the top of the centrifuged sample and stored at –80°C in the Odense Artery Biobank.

### DNA extraction, WES, and data analysis.

Genomic DNA was isolated from snap-frozen tissue samples and buffy coats. WES was conducted on matched buffy coat and tissue samples obtained from the 24 patients.

Genomic DNA was isolated from fresh-frozen tissue samples and buffy coats using standard methods. The libraries were prepared using 50 ng DNA and a Twist Comprehensive Exome Panel kit (Twist Bioscience), and 2 × 150 bp paired-end sequencing was performed on an Illumina NovaSeq 6000 platform. Unique dual indexing was applied to reduce cross-sample contamination from index hopping. Furthermore, unique molecular identifiers (UMIs) from IDT were applied for error suppression.

Using Illumina DRAGEN, sequencing reads were UMI-collapsed with error correction and subsequently aligned. We sequenced both matched buffy coat and tissue samples at 700× coverage to be able to identify with high confidence somatic variants and distinguish them from sequencing artifacts, even at lower VAFs (see somatic variant calling approach below). We set the lowest VAF at 1%, and discarded variants with under 5 reads. Mutations identified in 2 independent sequencing rounds (starting from raw DNA sample) from the same patient were included in the subsequent analyses. The mutations presented in the results are those that were consistently identified in more than one independent sequencing for each plaque. To determine the clonal cell frequencies, we calculated them from VAFs obtained in both sequencing rounds, while taking into account the number of reads per sequencing round. Clonal cell frequencies were calculated based on the assumption of diploid cells and mutation heterozygosity by multiplying VAFs by 2, while VAFs of mutations located on the X-chromosome in male patients was multiplied by 1.

In the first analysis, somatic mutations specific to tissue samples (plaques, ATAs, and ITAs) were identified using Mutect2 (https://gatk.broadinstitute.org/hc/en-us/articles/9570422171291-Mutect2) joint tumor-normal variant calling, with patient-matched buffy coat DNA serving as the normal reference.

In the second analysis, buffy coat DNA samples were analyzed to identify carriers of CHIP somatic mutations within the hematopoietic system. Using a set of 78 previously established CHIP genes (10), we applied Mutect2 in tumor-only mode to the buffy coat sequencing data. We then assessed the presence of these identified CHIP mutations in tissue samples from the same individual, testing the hypothesis that hematopoietic cells carrying CHIP mutations infiltrate plaque tissue. In addition, to ensure that none of the identified CHIP mutations was caused by platform-specific noise, all found mutations were force-called across all samples in all patients. Mutations that appeared in more than one patient were excluded.

For both the tumor-normal and tumor-only analyses, germline mutations (having a VAF of approximately 50% in all samples from a patient) were removed, and common single-nucleotide polymorphisms were removed by filtering against a panel of normal generated by the 1000 Genomes Project samples, and by using population allele frequencies of common and rare variants from the Genome Aggregation Database (gnomAD) (https://console.cloud.google.com/storage/browser/gatk-best-practices/).

Variants were annotated using VarSeq version 2.2.1 (Golden Helix, Inc.) and filtered through a cascade of filters. Briefly, for variant calling in non-matched samples, the filters were (a) variant is in a CHIP gene according to our list of 78 CHIP genes (10), (b) the region is covered by at least 20 reads, (c) has a known or predicted damaging effect on protein functionality (according to ClinVar or prediction tools), and (d) has a minor allele frequency (MAF) of less than 1% (according to gnomAD). gnomAD is the world’s largest public collection of genetic variation, and it is based on 125,748 exomes and 15,708 whole genomes collected worldwide (https://gnomad.broadinstitute.org/). We used the gnomAD collection from exome and genome sequencing data as a large external control group. For matched Mutect2 calling tissue versus buffy coat, we selected variants with (a) at least 5 reads, (b) a VAF in reads of 1% or greater, and no or very low signal in the buffy coat (<0.01%), to eliminate variants that are present also in the buffy coat and were erroneously included by the variant calling process. Built-in postcalling filters in Mutect2 were used to select for high-quality variants. A base quality threshold of 25 was set to accept a variant. All filtered variants were inspected manually using VarSeq; mapping artifacts and low-quality calls were removed.

For functional prediction of nonsynonymous variants, we used the Combined Annotation Dependent Depletion (CADD Scores 1.4) method (PHRED scaled scores) (44). The CADD algorithm includes conservation metrics, functional genomic data, exon-intron boundaries, and protein functionality scores. A score of 20 or greater indicates that a variant is predicted to be among the 1% of the most deleterious substitutions. We used a CADD score of over 23 to select for a damaging effect. In addition, we used a dbNSFP tool (database for nonsynonymous single nucleotide polymorphisms’ functional predictions), which incorporates 5 different algorithms (SIFT, Polyphen2, MutationTaster, MutationAssessor, and FATHMM) (45). The most damaging variants were selected based on a voting system where 4 or all 5 of these algorithms predicted a negative functional impact.

### ddPCR.

The QX200 Droplet Digital system from Bio-Rad was used for ddPCR analysis and mutation detection assays were ordered from Bio-Rad. Mutation detection assays contained primers and 2 TaqMan hydrolysis probes capable of binding either the wild-type or mutated sequence. Genomic DNA from plaque tissue and buffy coat was preamplified by a 10-cycle PCR reaction nesting the Bio-Rad assay primers using Q5 High-Fidelity DNA polymerase (M0491L, New England BioLabs). While the sequences of the primers and probes were proprietary to Bio-Rad, we obtained information regarding the amplicon size (*x* bp) of the Bio-Rad primers. To ensure compatibility, preamplification primers were designed to produce an amplicon with at least *x* bp flanking the mutation on either side, ensuring the Bio-Rad primer binding sites were present within the preamplification product (details on mutations and sequences are provided in Supplemental Table 1).

Preamplified amplicons were diluted 1:10 or 1:100 before proceeding with ddPCR.

Diluted preamplified DNA was mixed with mutation detection assays (TaqMan hydrolysis probes and primers), ddPCR Multiplex Supermix (12005909, Bio-Rad), 300 mM DTT, and nuclease-free water. Samples were partitioned to water-in-oil droplets using a QX200 Droplet Generator (1864002, Bio-Rad) and then carefully transferred to a ddPCR 96-Well Plate (12001925, Bio-Rad). Partitioned samples underwent a 40-cycle PCR and were kept at 4°C overnight to enhance droplet counts. A QX200 Droplet Reader (1864003, Bio-Rad) was used to read the endpoint fluorescence signal in droplets and data were analyzed with QX Manager software version 2.1 (Bio-Rad).

### Histology and immunohistochemistry.

Formalin-fixed, paraffin-embedded tissue blocks were used for histology and immunohistochemistry. For Masson’s trichome staining, sections were deparaffinized and exposed to Papaniculaus 1 (6 minutes), running tap water (8 minutes), 1.2% picric acid (5 minutes), tap water (1 minute), 1% Biebrick scarlet (10 minutes), 1% phosphor wolfram acid (10 minutes), 2.5% methyl blue (2 minutes), acetic acid (30 seconds), 1% phosphor wolfram acid (5 minutes), and acetic acid (3 minutes). Slides were then dehydrated, and coverslips were mounted using Aquatex (Sigma-Aldrich). For Oil Red O staining, sections were exposed to 7% ethanol (1 minute), Oil Red O (2.5 minutes), 100% ethanol (1 minute), tap water (1 minute), Mayer’s hematoxylin (2 minutes), tap water (1 minute), 0.3% sodium carbonate (10 seconds), and running tap water (1 minute). Coverslips were then mounted using Aquatex (Sigma-Aldrich). CD68 immunohistochemistry was performed at the Department of Pathology, OUH, using primary antibody PG-M1 (M0876, Dako: 1:50) utilizing the fully automated OptiView DAB IHC Detection Kit (760-700, Roche). Images were recorded by a Hamamatsu model 2 OHT scanner.

### Biobank of Karolinska Endarterectomies.

Patients undergoing surgery for symptomatic or asymptomatic, high-grade (>50% NASCET) (46) carotid stenosis at the Department of Vascular Surgery, Karolinska University Hospital and Department of Surgery, Vascular section, Södersjukhuset, Stockholm, Sweden, were enrolled in the study and clinical data recorded on admission. Symptoms of plaque instability were defined as a transitory ischemic attack, minor stroke, and amaurosis fugax. Patients without qualifying symptoms within 6 months prior to surgery were categorized as asymptomatic and indication for carotid endarterectomy was based on results from the Asymptomatic Carotid Surgery Trial (ACST) (47). Carotid plaques and blood samples were collected at surgery and retained within the Biobank of Karolinska Endarterectomies (BiKE). The BiKE study cohort demographics, details of sample collection, processing, and large-scale analyses (genotyping, transcriptomic and proteomic profiling) were as previously described (48–51). For microarrays, plaques (*n* = 127) were divided transversally at the most stenotic part and the proximal half of the lesion was used for RNA preparation, while the distal half was fixed in 4% Zn-formaldehyde and processed for histology. Normal artery controls were obtained from 9 macroscopically disease-free iliac arteries and 1 aorta from organ donors without a history of cardiovascular disease.

### Statistics.

Gene ontology enrichment analyses presented in Figure 3, A and B were conducted with the clusterProfiler 4.0 package (52) in R using the default enrichGO function (ont = “ALL”, pAdjustMethod = “BH”, pvalueCutoff = 0.05, qvalueCutoff = 0.05). The correlation analyses shown in Figure 1H and Figure 4C were performed using Spearman’s rank correlation coefficient.

Comparisons of mean expression within each cell cluster in Figure 3C and Supplemental Figures 4 and 5 were conducted by Mann-Whitney *U* test. A *P* value of less than 0.05 was considered significant.

### Study approval.

The study was approved by the Danish National Committee on Health Research Ethics (project ID: CVK-2006749) and The Regional Committees on Health Research Ethics for Southern Denmark (project ID: S-20140202) and is registered in the Registry of Research Projects in the Region of Southern Denmark (file no. 18/20280). Written informed consent was obtained from all participants prior to surgery. The investigation conformed with the principles outlined in the Declaration of Helsinki.

### BiKE ethics.

All BiKE human samples were collected with written informed consent from patients or organ donors’ guardians. BiKE studies were approved by the regional Ethical Committee and followed the guidelines of the Declaration of Helsinki.

### Data availability.

WES data generated and analyzed during the current study are not publicly available due to hospital guidelines and legislation regarding personal data by the European General Data Protection Regulation (GDPR). Data will be available from the corresponding author on reasonable request and with permission from the OUH Legal Department.

The full microarray data from the BiKE cohort have been deposited at the NCBI Gene Expression Omnibus and are publicly available (GEO GSE21545). The individual human data cannot be deposited or shared because of the GDPR and ethics laws that regulate the privacy of individuals who participated in the study. Other data reported in this paper on the group level may be shared upon a reasonable request.

Values for all figures are supplied as a supplemental Supporting Data Values file.

## Author contributions

LBS, MT, and LMR designed the study. LBS, JSL, and LMR acquired funding for the study. JSL, KCH, LPR, UH, and LM provided patient samples. LBS, SK, PSJ, MKP, UH, and LM conducted the experiments and acquired the data. SMB, MJL, MD, LVBA, SK, LBS, MKP, LM, and OB analyzed the data. LBS, SK, and LMR wrote the manuscript. All authors revised and approved the final manuscript.

## Supplementary Material

Supplemental data

Supporting data values

## Version 1 04/08/2025

In-Press Preview

## Version 2 05/22/2025

Electronic publication
