# Supplementary material for: Mutational landscape of atherosclerotic plaques reveals large clonal cell populations
Source: JCI Insight. 2025 Apr 8;10(10):e188281. doi: 10.1172/jci.insight.188281 (PMC12128991; doi:10.1172/jci.insight.188281)
Supplement: Supplemental data [file jciinsight-10-188281-s181.pdf]

Supplemental Table S1

gene	mutation	position	mutation effect	pre-amplification forward primer	pre-amplification reverse primer	amplicon size (bp) of pre-amplification primers	amplicon size (bp) of Bio-Rad primers (sequences are intellectual property of Bio-Rad)
BAZ1A	G > C	14:34776440	missense	AAAAGCACCACCGATTTGGC	ACTTGGGGTAACTTTGTATCTCA	529	67
UTP20	C > T	12:101357071	missense	TGAAAGTGTGTGCCCTACTCA	TACTGCTCAGAGTGTCAGTGGA	274	80
ARHGAP36	C > T	X:131058430	missense	CGACGCAAAGGTTAACTGCG	ACGACTCTAGGGGGTCCATT	578	62
DSG1	G > A	18:31338418	missense	CATTCCATCAGAAATGGGTGCT	TCCTTTCAGCTCAACTATGCT	755	78
HGD	A > T	3:120644421	synonymous/non-cds	GCAAACAGCTTGCCCTGGTA	TCATGCAACCATGGGCATCT	185	64
OPN4	C > T	10:86663859	synonymous/non-cds	GGCATGCACAATTTACGGGG	CTCGCAGTCACACAGAGAGG	831	65
TET2	G > T	4:105269613	loss-of-function	TAGCATACTTTATGGCCTCAATAAC	CTGTCCTCAGCCCAACTTAC	370	116

Supplemental Table S2

	Carotid plaque donors (carotid endarterectomy)	ATA and ITA donors (coronary bypass)	*p**
**patients, *n***	**13**	**11**	
male sex, *n* (%)	8 (62%)	8 (73%)	0.562
age at surgery, years (mean ± sd)	75.8 ± 6.2	67.1 ± 6.2	0.003
body-mass index, kg/m^2 (mean ± sd)	26 ± 6.4	29 ± 3.2 (*n* = 10)	0.082
systolic blood pressure, mmHg (mean ± sd)	146 ± 15.9	139 ± 15.8 (*n* = 10)	0.307
diastolic blood pressure, mmHg (mean ± sd)	79 ± 13.7	73 ± 11.0 (*n* = 10)	0.256
high-density lipoprotein, mmol/L (mean ± sd)	1.2 ± 0.3 (*n* = 11)	1.1 ± 0.3 (*n* = 10)	0.459
low-density lipoprotein, mmol/L (mean ± sd)	2.5 ± 1.4 (*n* = 11)	2.0 ± 0.8 (*n* = 10)	0.285
triglycerides, mmol/L (mean ± sd)	1.83 ± 0.92 (*n* = 9)	1.78 ± 1.0 (*n* = 10)	0.922
smoking, active/former+never/NA	6/5/2	3/7/1	0.470
diabetes mellitus, yes/no/NA	2/5/6	3/7/1	0.138
statins, yes/no	9/4	9/2	0.478

*NA = not available; * Student's t-test for continuous variables and Chi-squared test for categorical variables.*

Supplemental Table S3

patient	sample	chromosomal position	gene	reference / mutation	effect	clone cell frequency
1	1	10:5212712	*AKR1C4*	C/T	loss of function	0.058
1	1	11:4367352	*OR52B4*	T/C	synonymous or non-coding sequence	0.046
1	1	12:110486579	*FAM216A*	A/-	loss of function	0.044
1	1	13:101081551	*NALCN*	C/A	missense	0.034
1	1	16:56670648	*MT1H*	-/GTGCTT	synonymous or non-coding sequence	0.033
1	1	18:78994342	*SALL3*	C/T	missense	0.041
1	1	4:118313225	*PRSS12*	C/T	missense	0.037
1	1	6:110246023	*METTL24*	G/A	loss of function	0.038
1	1	X:108732740	*IRS4*	T/A	missense	0.023
1	2	X:108732740	*IRS4*	T/A	missense	0.034
1	1	1:85094505	*DNAI3*	C/G	missense	0.053
1	1	10:43387246	*HNRNPF*	G/A	synonymous or non-coding sequence	0.040
1	1	10:86663859	*OPN4*	C/T	synonymous or non-coding sequence	0.086
1	1	11:1196038	*MUC5AC*	G/A	synonymous or non-coding sequence	0.040
1	1	12:57162929	*LRP1*	C/T	loss of function	0.064
1	1	15:89645099	*KIF7*	C/T	missense	0.044
1	1	17:44862804	*EFTUD2*	G/C	missense	0.044
1	1	19:1044708	*ABCA7*	G/-	loss of function	0.066
1	1	2:79121595	*REG1A*	G/A	missense	0.053
1	1	20:43540286	*L3MBTL1*	T/A	missense	0.051
1	1	4:26428792	*RBPJ*	G/A	missense	0.029
1	1	4:49044789	*CWH43*	A/T	missense	0.039
1	1	5:146340146	*POU4F3*	A/G	missense	0.082
1	1	6:72097009	*RIMS1*	G/A	synonymous or non-coding sequence	0.055
1	1	7:127373670	*ZNF800*	A/C	missense	0.078
1	1	7:38742056	*VPS41*	T/C	missense	0.046
1	1	7:43623859	*STK17A*	C/G	missense	0.064
1	2	8:24463958	*ADAM7*	A/G	missense	0.092
1	3	1:6097439	*KCNAB2*	G/A	synonymous or non-coding sequence	0.063
1	3	11:78726136	*TENM4*	C/T	missense	0.055
1	3	14:35402821	*NFKBIA*	G/A	synonymous or non-coding sequence	0.054
1	3	14:92134253	*CPSF2*	C/T	loss of function	0.065
1	3	16:67256477	*SLC9A5*	T/G	missense	0.076
1	3	18:26916653	*CHST9*	T/C	missense	0.060
1	3	19:8871628	*MUC16*	G/A	missense	0.053
1	3	2:219038510	*CFAP65*	G/C	synonymous or non-coding sequence	0.057
1	3	21:26837864	*ADAMTS1*	A/C	synonymous or non-coding sequence	0.062
1	3	5:41048348	*MROH2B*	G/A	missense	0.074
1	3	7:138752720	*ATP6V0A4*	A/G	missense	0.073
1	3	8:24463958	*ADAM7*	A/G	missense	0.083
1	4	1:154544506	*TDRD10*	C/T	synonymous or non-coding sequence	0.061
1	4	1:167434071	*CD247*	C/T	synonymous or non-coding sequence	0.088
1	4	1:169540536	*F5*	A/G	synonymous or non-coding sequence	0.093
1	4	1:245602780	*KIF26B*	T/C	synonymous or non-coding sequence	0.115
1	4	1:85094505	*DNAI3*	C/G	missense	0.027
1	4	10:102450666	*C10orf95*	C/A	missense	0.163
1	4	10:20897199	*NEBL*	C/T	missense	0.114
1	4	10:86663859	*OPN4*	C/T	synonymous or non-coding sequence	0.306
1	4	11:100070482	*CNTN5*	C/T	synonymous or non-coding sequence	0.084
1	4	11:1162614	*MUC5AC*	G/T	missense	0.101
1	4	11:119638762	*NECTIN1*	C/G	missense	0.043
1	4	11:128937795	*TP53AIP1*	G/T	missense	0.030
1	4	11:128937796	*TP53AIP1*	C/G	missense	0.030
1	4	11:62649155	*INTS5*	T/A	missense	0.041
1	4	11:78726136	*TENM4*	C/T	missense	0.031
1	4	12:12330386	*MANSC1*	A/G	missense	0.123
1	4	12:2666736	*CACNA1C*	C/G	missense	0.062
1	4	12:51888673	*ANKRD33*	G/A	missense	0.065
1	4	12:55748855	*GDF11*	G/C	missense	0.072
1	4	12:57162929	*LRP1*	C/T	loss of function	0.203
1	4	12:63600318	*DPY19L2*	G/A	synonymous or non-coding sequence	0.133
1	4	13:113094599	*MCF2L*	C/T	synonymous or non-coding sequence	0.038
1	4	14:20391611	*TEP1*	G/A	synonymous or non-coding sequence	0.077
1	4	14:55433712	*TBPL2*	C/T	missense	0.042
1	4	14:64487032	*ZBTB25*	G/C	missense	0.079
1	4	15:26548002	*GABRB3*	G/C	missense	0.144
1	4	15:28272385	*HERC2*	G/C	missense	0.092
1	4	15:33848350	*RYR3*	C/T	synonymous or non-coding sequence	0.094
1	4	15:89645099	*KIF7*	C/T	missense	0.046
1	4	16:68274888	*SLC7A6*	G/A	synonymous or non-coding sequence	0.024
1	4	16:76316408	*CNTNAP4*	G/T	synonymous or non-coding sequence	0.070
1	4	17:63688556	*MAP3K3*	G/C	missense	0.112
1	4	17:78398291	*PGS1*	GCAAAGTTTCCTTCA	loss of function	0.040

1	4	18:26916653	*CHST9*	T/C	missense	0.035
1	4	18:79344352	*ATP9B*	G/C	missense	0.117
1	4	19:17787741	*FCHO1*	G/A	missense	0.039
1	4	19:35723120	*KMT2B*	C/T	missense	0.035
1	4	19:41016662	*CYP2B6*	T/C	synonymous or non-coding sequence	0.029
1	4	19:48497426	*LMTK3*	C/T	missense	0.040
1	4	2:165876167	*TTC21B*	G/A	loss of function	0.097
1	4	2:219038510	*CFAP65*	G/C	synonymous or non-coding sequence	0.028
1	4	2:227251191	*COL4A3*	C/G	missense	0.068
1	4	2:26204109	*HADHA*	ATCT/-	loss of function	0.103
1	4	2:26204113	*HADHA*	T/G	missense	0.104
1	4	2:37951493	*RMDN2*	A/T	missense	0.052
1	4	2:96877905	*FAM178B*	G/T	synonymous or non-coding sequence	0.064
1	4	2:98300153	*VWA3B*	A/G	synonymous or non-coding sequence	0.088
1	4	20:46045922	*SLC12A5*	G/A	synonymous or non-coding sequence	0.062
1	4	20:57564505	*PCK1*	T/C	missense	0.084
1	4	21:14966436	*NRIP1*	G/C	missense	0.078
1	4	3:113581388	*SIDT1*	G/A	missense	0.099
1	4	3:78617987	*ROBO1*	G/C	synonymous or non-coding sequence	0.070
1	4	4:109760595	*CFI*	A/G	missense	0.083
1	4	4:150867694	*LRBA*	T/C	missense	0.024
1	4	4:17515390	*CLRN2*	A/G	missense	0.029
1	4	4:49044789	*CWH43*	A/T	missense	0.042
1	4	5:140654027	*IK*	G/C	missense	0.041
1	4	5:146340146	*POU4F3*	A/G	missense	0.265
1	4	5:90647720	*ADGRV1*	C/T	missense	0.120
1	4	6:137871507	*TNFAIP3*	G/A	missense	0.048
1	4	6:137877089	*TNFAIP3*	T/G	synonymous or non-coding sequence	0.081
1	4	6:158501918	*TULP4*	A/G	missense	0.093
1	4	6:39057536	*GLP1R*	C/T	synonymous or non-coding sequence	0.126
1	4	6:43132774	*PTK7*	C/T	synonymous or non-coding sequence	0.035
1	4	7:127373670	*ZNF800*	A/C	missense	0.231
1	4	7:138752720	*ATP6V0A4*	A/G	missense	0.025
1	4	7:38742056	*VPS41*	T/C	missense	0.028
1	4	7:43623859	*STK17A*	C/G	missense	0.117
1	4	7:6130285	*USP42*	C/T	synonymous or non-coding sequence	0.034
1	4	8:131040178	*ADCY8*	G/A	synonymous or non-coding sequence	0.078
1	4	8:144080120	*EXOSC4*	C/T	missense	0.077
1	4	8:144096359	*CYC1*	A/G	missense	0.084
1	4	8:22513420	*PPP3CC*	C/T	missense	0.139
1	4	X:136511796	*HTATSF1*	C/G	missense	0.018
1	4	X:136548831	*VGLL1*	C/T	missense	0.104
1	4	X:312872	*GTPBP6*	C/G	missense	0.050
1	5	1:154544506	*TDRD10*	C/T	synonymous or non-coding sequence	0.058
1	5	1:167434071	*CD247*	C/T	synonymous or non-coding sequence	0.040
1	5	1:169540536	*F5*	A/G	synonymous or non-coding sequence	0.062
1	5	1:6097439	*KCNAB2*	G/A	synonymous or non-coding sequence	0.040
1	5	10:86663859	*OPN4*	C/T	synonymous or non-coding sequence	0.096
1	5	11:100070482	*CNTN5*	C/T	synonymous or non-coding sequence	0.047
1	5	11:119638762	*NECTIN1*	C/G	missense	0.086
1	5	11:128937795	*TP53AIP1*	G/T	missense	0.071
1	5	11:128937796	*TP53AIP1*	C/G	missense	0.071
1	5	11:62649155	*INTS5*	T/A	missense	0.079
1	5	11:78726136	*TENM4*	C/T	missense	0.079
1	5	12:12330386	*MANSC1*	A/G	missense	0.031
1	5	12:55748855	*GDF11*	G/C	missense	0.051
1	5	12:57162929	*LRP1*	C/T	loss of function	0.078
1	5	12:6843422	*GNB3*	G/A	synonymous or non-coding sequence	0.046
1	5	13:113094599	*MCF2L*	C/T	synonymous or non-coding sequence	0.058
1	5	14:35402821	*NFKBIA*	G/A	synonymous or non-coding sequence	0.027
1	5	14:55433712	*TBPL2*	C/T	missense	0.070
1	5	14:92134253	*CPSF2*	C/T	loss of function	0.095
1	5	15:28272385	*HERC2*	G/C	missense	0.068
1	5	15:33848350	*RYR3*	C/T	synonymous or non-coding sequence	0.060
1	5	15:45163646	*DUOX1*	A/G	missense	0.033
1	5	16:67256477	*SLC9A5*	T/G	missense	0.040
1	5	16:68274888	*SLC7A6*	G/A	synonymous or non-coding sequence	0.065
1	5	16:76316408	*CNTNAP4*	G/T	synonymous or non-coding sequence	0.069
1	5	18:26916653	*CHST9*	T/C	missense	0.101
1	5	19:17787741	*FCHO1*	G/A	missense	0.066
1	5	19:2761490	*SGTA*	G/C	synonymous or non-coding sequence	0.049
1	5	19:30444261	*ZNF536*	C/A	synonymous or non-coding sequence	0.081
1	5	19:30710888	*ZNF536*	T/C	missense	0.048
1	5	19:35723120	*KMT2B*	C/T	missense	0.073

1	5	19:41016662	*CYP2B6*	T/C	synonymous or non-coding sequence	0.048
1	5	19:48497426	*LMTK3*	C/T	missense	0.063
1	5	19:58480549	*ZNF446*	C/T	synonymous or non-coding sequence	0.058
1	5	19:8871628	*MUC16*	G/A	missense	0.059
1	5	2:134718768	*TMEM163*	C/G	missense	0.043
1	5	2:219038510	*CFAP65*	G/C	synonymous or non-coding sequence	0.053
1	5	2:227251191	*COL4A3*	C/G	missense	0.045
1	5	2:26204109	*HADHA*	ATCT/-	loss of function	0.059
1	5	2:26204113	*HADHA*	T/G	missense	0.059
1	5	2:37951493	*RMDN2*	A/T	missense	0.056
1	5	2:79121595	*REG1A*	G/A	missense	0.037
1	5	2:98300153	*VWA3B*	A/G	synonymous or non-coding sequence	0.040
1	5	20:43540286	*L3MBTL1*	T/A	missense	0.038
1	5	20:46045922	*SLC12A5*	G/A	synonymous or non-coding sequence	0.033
1	5	20:57564505	*PCK1*	T/C	missense	0.051
1	5	21:14966436	*NRIP1*	G/C	missense	0.040
1	5	21:26837864	*ADAMTS1*	A/C	synonymous or non-coding sequence	0.072
1	5	22:35546999	*RASD2*	G/T	missense	0.038
1	5	3:169812441	*LRRC34*	A/G	synonymous or non-coding sequence	0.029
1	5	3:78617987	*ROBO1*	G/C	synonymous or non-coding sequence	0.060
1	5	4:109760595	*CFI*	A/G	missense	0.046
1	5	4:150867694	*LRBA*	T/C	missense	0.093
1	5	4:17515390	*CLRN2*	A/G	missense	0.058
1	5	4:26428792	*RBPJ*	G/A	missense	0.054
1	5	4:49044789	*CWH43*	A/T	missense	0.043
1	5	5:140654027	*IK*	G/C	missense	0.057
1	5	5:146340146	*POU4F3*	A/G	missense	0.104
1	5	5:41048348	*MROH2B*	G/A	missense	0.081
1	5	6:158501918	*TULP4*	A/G	missense	0.050
1	5	6:43132774	*PTK7*	C/T	synonymous or non-coding sequence	0.069
1	5	6:72097009	*RIMS1*	G/A	synonymous or non-coding sequence	0.046
1	5	7:127373670	*ZNF800*	A/C	missense	0.081
1	5	7:138752720	*ATP6V0A4*	A/G	missense	0.036
1	5	7:2572257	*IQCE*	G/A	missense	0.036
1	5	7:38742056	*VPS41*	T/C	missense	0.033
1	5	7:43623859	*STK17A*	C/G	missense	0.101
1	5	7:5064101	*RBAK,RBAK-RL*	C/T	synonymous or non-coding sequence	0.052
1	5	7:6130285	*USP42*	C/T	synonymous or non-coding sequence	0.058
1	5	8:131040178	*ADCY8*	G/A	synonymous or non-coding sequence	0.031
1	5	8:144080120	*EXOSC4*	C/T	missense	0.040
1	5	8:144096359	*CYC1*	A/G	missense	0.051
1	5	8:71843707	*MSC*	G/A	missense	0.036
1	5	9:110496887	*SVEP1*	C/T	synonymous or non-coding sequence	0.059
1	5	9:72059313	*C9orf57*	C/G	missense	0.038
1	5	X:101292846	*TAF7L*	G/A	missense	0.022
1	5	X:105267517	*IL1RAPL2*	C/T	loss of function	0.033
1	5	X:130066909	*ELF4*	G/A	missense	0.046
1	5	X:136511796	*HTATSF1*	C/G	missense	0.019
1	5	X:40105901	*BCOR*	G/A	synonymous or non-coding sequence	0.031
1	6	4:26428792	*RBPJ*	G/A	missense	0.045
1	7	1:245602780	*KIF26B*	T/C	synonymous or non-coding sequence	0.054
1	7	10:86663859	*OPN4*	C/T	synonymous or non-coding sequence	0.050
1	7	11:1162614	*MUC5AC*	G/T	missense	0.049
1	7	12:12330386	*MANSC1*	A/G	missense	0.042
1	7	12:57162929	*LRP1*	C/T	loss of function	0.052
1	7	15:26548002	*GABRB3*	G/C	missense	0.038
1	7	16:4695136	*NUDT16L1*	A/T	missense	0.037
1	7	16:55485337	*MMP2*	C/T	missense	0.041
1	7	17:63688556	*MAP3K3*	G/C	missense	0.038
1	7	17:81869003	*ARHGDIA*	T/C	missense	0.040
1	7	18:79344352	*ATP9B*	G/C	missense	0.037
1	7	4:26428792	*RBPJ*	G/A	missense	0.053
1	7	5:141414811	*PCDHGA1,PCD*	C/G	missense	0.038
1	7	5:146340146	*POU4F3*	A/G	missense	0.062
1	7	6:30915220	*VARS2*	A/C	missense	0.037
1	7	7:127373670	*ZNF800*	A/C	missense	0.052
1	7	8:22513420	*PPP3CC*	C/T	missense	0.029
1	7	9:128797124	*TBC1D13*	C/G	synonymous or non-coding sequence	0.042
1	7	X:136548831	*VGLL1*	C/T	missense	0.021
1	8	4:26428792	*RBPJ*	G/A	missense	0.087
1	9	4:26428792	*RBPJ*	G/A	missense	0.036
2	1	11:33645756	*KIAA1549L*	G/A	missense	0.029
2	1	13:36437812	*CCNA1*	G/A	missense	0.024
2	1	18:10705692	*PIEZO2*	G/A	synonymous or non-coding sequence	0.030

2	1	19:49070542	*KCNA7*	G/A	synonymous or non-coding sequence	0.034
2	1	19:53166017	*ZNF665*	T/G	missense	0.045
2	1	19:57830545	*ZNF587B*	C/T	missense	0.022
2	1	2:24039004	*WDCP*	C/A	missense	0.032
2	1	21:39661044	*B3GALT5*	A/G	missense	0.040
2	1	5:131521439	*RAPGEF6*	C/G	missense	0.042
2	1	7:130276700	*CPA2*	G/A	missense	0.027
2	1	8:122953849	*ZHX2*	A/C	missense	0.033
2	1	X:111727377	*ALG13*	A/C	missense	0.042
2	2	1:109340771	*SORT1*	T/C	missense	0.072
2	2	1:151133357	*SEMA6C*	G/A	synonymous or non-coding sequence	0.098
2	2	1:204618067	*LRRN2*	C/T	synonymous or non-coding sequence	0.055
2	2	1:204618068	*LRRN2*	A/C	missense	0.055
2	2	1:248387973	*OR2T6*	G/T	missense	0.189
2	2	1:42430819	*ZMYND12*	C/T	missense	0.134
2	2	1:44118986	*KLF17*	C/T	loss of function	0.087
2	2	10:79352353	*PPIF*	A/G	missense	0.157
2	2	11:4999679	*OR51L1*	C/T	missense	0.096
2	2	12:8224096	*FAM90A1*	G/A	synonymous or non-coding sequence	0.089
2	2	13:39039231	*NHLRC3*	C/G	synonymous or non-coding sequence	0.141
2	2	14:23416067	*MYH7*	G/A	synonymous or non-coding sequence	0.100
2	2	14:55637331	*KTN1*	G/A	synonymous or non-coding sequence	0.229
2	2	15:43060057	*UBR1*	T/C	missense	0.101
2	2	15:74892706	*MPI*	G/A	missense	0.159
2	2	17:40443926	*IGFBP4*	G/T	missense	0.068
2	2	18:58330802	*NEDD4L*	C/G	missense	0.060
2	2	19:15807788	*OR10H1*	C/T	missense	0.073
2	2	19:18532175	*FKBP8*	C/G	missense	0.129
2	2	19:57753404	*ZNF776*	T/G	missense	0.172
2	2	2:159748000	*MARCHF7*	A/G	missense	0.206
2	2	22:22549936	*PRAME*	G/T	missense	0.107
2	2	22:32445852	*BPIFC*	G/A	synonymous or non-coding sequence	0.077
2	2	3:140466700	*CLSTN2*	G/A	missense	0.058
2	2	3:172636026	*NCEH1*	T/C	missense	0.108
2	2	4:104491144	*CXXC4*	C/T	missense	0.100
2	2	4:99621078	*MTTP*	C/A	missense	0.101
2	2	6:10586367	*GCNT2*	T/C	synonymous or non-coding sequence	0.159
2	2	6:3154570	*TUBB2A*	A/C	missense	0.185
2	2	6:43624688	*GTPBP2*	T/G	missense	0.172
2	2	7:101128633	*SERPINE1*	G/T	missense	0.176
2	2	8:143867936	*EPPK1*	G/A	missense	0.089
2	2	8:60742058	*CHD7*	G/A	missense	0.086
2	2	8:60742059	*CHD7*	G/T	missense	0.086
2	2	8:638382	*ERICH1*	G/A	synonymous or non-coding sequence	0.092
2	3	1:109322934	*SORT1*	G/C	synonymous or non-coding sequence	0.057
2	3	1:248387973	*OR2T6*	G/T	missense	0.023
2	3	11:2402862	*TSSC4*	G/A	missense	0.044
2	3	11:33645756	*KIAA1549L*	G/A	missense	0.075
2	3	12:108799053	*SSH1*	G/C	synonymous or non-coding sequence	0.088
2	3	12:108799056	*SSH1*	GCGCGTGATGCT/-	missense	0.081
2	3	12:113007878	*OAS2*	G/A	synonymous or non-coding sequence	0.074
2	3	12:121827752	*SETD1B*	C/G	synonymous or non-coding sequence	0.047
2	3	12:129074304	*TMEM132D*	C/-	loss of function	0.085
2	3	12:55027319	*NEUROD4*	C/T	missense	0.052
2	3	13:36437812	*CCNA1*	G/A	missense	0.059
2	3	14:105731985	*intergenic*	G/A	synonymous or non-coding sequence	0.120
2	3	15:84944765	*SLC28A1*	A/G	missense	0.108
2	3	17:41757649	*JUP*	G/A	missense	0.100
2	3	17:76010360	*EVPL*	G/A	missense	0.054
2	3	17:7923060	*KCNAB3*	GGGTGCA/-	synonymous or non-coding sequence	0.107
2	3	18:10705692	*PIEZO2*	G/A	synonymous or non-coding sequence	0.064
2	3	19:39101506	*ACP7*	G/A	missense	0.058
2	3	19:48724509	*RASIP1*	C/A	missense	0.047
2	3	19:49070542	*KCNA7*	G/A	synonymous or non-coding sequence	0.046
2	3	19:57830545	*ZNF587B*	C/T	missense	0.037
2	3	2:131363582	*RAB6D*	C/T	missense	0.132
2	3	2:24039004	*WDCP*	C/A	missense	0.032
2	3	20:25425214	*GINS1*	G/A	missense	0.067
2	3	3:10177950	*IRAK2*	C/G	synonymous or non-coding sequence	0.063
2	3	3:160404400	*SMC4*	TCTG/-	loss of function	0.098
2	3	3:184305807	*PSMD2*	G/A	missense	0.041
2	3	3:47002055	*NBEAL2*	T/A	missense	0.038
2	3	4:154585611	*FGA*	C/G	missense	0.033
2	3	4:55607309	*NMU*	A/T	missense	0.050

2	3	5:131521439	*RAPGEF6*	C/G	missense	0.068
2	3	5:160622458	*ATP10B*	A/G	missense	0.106
2	3	5:17498642	*intergenic*	G/A	synonymous or non-coding sequence	0.115
2	3	5:177524503	*FAM193B*	C/A	loss of function	0.084
2	3	5:177524506	*FAM193B*	G/-	loss of function	0.084
2	3	6:31727593	*DDAH2*	G/A	missense	0.090
2	3	7:101128633	*SERPINE1*	G/T	missense	0.065
2	3	7:130276700	*CPA2*	G/A	missense	0.049
2	3	7:135625318	*NUP205*	C/T	missense	0.043
2	3	8:122953849	*ZHX2*	A/C	missense	0.057
2	3	8:24918003	*NEFM*	G/A	synonymous or non-coding sequence	0.106
2	3	9:125241052	*HSPA5*	C/T	synonymous or non-coding sequence	0.089
2	3	9:127749474	*SH2D3C*	G/T	missense	0.032
2	4	2:209819137	*UNC80*	T/C	missense	0.028
2	5	2:209819137	*UNC80*	T/C	missense	0.049
2	5	21:43094734	*U2AF1*	A/T	missense	0.040
2	5	7:139454158	*KLRG2*	G/T	synonymous or non-coding sequence	0.064
2	6	1:109322934	*SORT1*	G/C	synonymous or non-coding sequence	0.064
2	6	1:13390351	*PRAMEF17*	C/T	missense	0.095
2	6	1:186090890	*HMCN1*	T/C	missense	0.073
2	6	1:46815101	*CYP4B1*	G/A	missense	0.107
2	6	11:134277267	*GLB1L3*	C/G	synonymous or non-coding sequence	0.100
2	6	11:28094780	*KIF18A*	C/T	missense	0.108
2	6	11:4682481	*OR51E2*	C/G	missense	0.054
2	6	11:66295001	*TMEM151A*	A/G	missense	0.056
2	6	14:105624659	*intergenic*	C/T	synonymous or non-coding sequence	0.040
2	6	16:2036438	*SLC9A3R2*	C/T	missense	0.110
2	6	17:20451827	*LGALS9B*	G/A	synonymous or non-coding sequence	0.049
2	6	19:12526887	*ZNF564*	T/A	synonymous or non-coding sequence	0.120
2	6	19:16436988	*EPS15L1*	G/C	missense	0.103
2	6	19:51769037	*FPR2*	G/A	missense	0.087
2	6	19:53166017	*ZNF665*	T/G	missense	0.071
2	6	19:54982999	*NLRP2*	G/T	missense	0.113
2	6	19:57830545	*ZNF587B*	C/T	missense	0.072
2	6	2:24039004	*WDCP*	C/A	missense	0.080
2	6	2:73224940	*SMYD5*	G/A	missense	0.094
2	6	20:63350730	*CHRNA4*	G/-	loss of function	0.128
2	6	21:39661044	*B3GALT5*	A/G	missense	0.084
2	6	3:138505346	*CEP70*	C/A	missense	0.102
2	6	3:184305807	*PSMD2*	G/A	missense	0.060
2	6	3:36528741	*STAC*	G/C	missense	0.091
2	6	3:49861012	*CAMKV*	T/C	missense	0.055
2	6	4:150906386	*LRBA*	T/C	missense	0.129
2	6	4:55607309	*NMU*	A/T	missense	0.101
2	6	5:177394037	*SLC34A1*	T/A	missense	0.078
2	6	6:31934388	*C2*	C/T	missense	0.113
2	6	9:96541861	*CDC14B*	T/A	missense	0.089
2	6	X:14008968	*GEMIN8*	T/A	missense	0.030
2	7	1:13390351	*PRAMEF17*	C/T	missense	0.077
2	7	1:46815101	*CYP4B1*	G/A	missense	0.027
2	7	11:134277267	*GLB1L3*	C/G	synonymous or non-coding sequence	0.090
2	7	16:2036438	*SLC9A3R2*	C/T	missense	0.026
2	7	19:51769037	*FPR2*	G/A	missense	0.021
2	7	19:53166017	*ZNF665*	T/G	missense	0.067
2	7	19:57830545	*ZNF587B*	C/T	missense	0.068
2	7	2:209819137	*UNC80*	T/C	missense	0.031
2	7	2:24039004	*WDCP*	C/A	missense	0.069
2	7	21:39661044	*B3GALT5*	A/G	missense	0.069
2	7	5:177394037	*SLC34A1*	T/A	missense	0.116
3	1	1:155133546	*EFNA1*	G/A	synonymous or non-coding sequence	0.039
3	1	19:5455821	*ZNRF4*	G/A	synonymous or non-coding sequence	0.030
3	1	6:159234384	*FNDC1*	C/T	missense	0.029
3	1	1:112724101	*TAFA3*	G/A	loss of function	0.083
3	1	1:1338372	*DVL1*	C/T	synonymous or non-coding sequence	0.039
3	1	1:44724548	*ARMH1*	G/A	synonymous or non-coding sequence	0.081
3	1	1:78013480	*DNAJB4*	T/G	missense	0.054
3	1	10:121790175	*ATE1*	C/T	missense	0.072
3	1	11:17611305	*OTOG*	C/T	missense	0.048
3	1	11:244462	*PSMD13*	G/A	missense	0.082
3	1	11:58943593	*GLYATL1*	G/A	synonymous or non-coding sequence	0.046
3	1	11:61743651	*DAGLA*	A/G	missense	0.046
3	1	11:64807196	*MEN1*	G/C	missense	0.070
3	1	12:102076827	*NUP37*	G/C	missense	0.047
3	1	12:5044970	*KCNA5*	T/C	missense	0.089

3	1	12:55932558	*DGKA*	T/C		synonymous or non-coding sequence	0.060
3	1	12:9101670	*A2M*	T/C		missense	0.057
3	1	14:91234391	*GPR68*	C/T		synonymous or non-coding sequence	0.031
3	1	16:31132296	*PRSS8*	G/A		missense	0.034
3	1	16:70398214	*ST3GAL2*	G/A		missense	0.037
3	1	16:89848989	*SPIRE2*	G/C		synonymous or non-coding sequence	0.085
3	1	17:10723284	*TMEM220*	G/A		synonymous or non-coding sequence	0.050
3	1	17:60209463	*USP32*	A/G		synonymous or non-coding sequence	0.069
3	1	17:61483238	*TBX4*	C/T		missense	0.089
3	1	17:7908010	*CHD3*	G/A		missense	0.035
3	1	18:63720865	*SERPINB11*	T/C		missense	0.041
3	1	18:743381	*YES1*	A/G		synonymous or non-coding sequence	0.057
3	1	19:21378029	*ZNF738*	A/C		synonymous or non-coding sequence	0.067
3	1	19:3590828	*GIPC3*	C/T		synonymous or non-coding sequence	0.048
3	1	2:111092935	*ACOXL*	C/T		missense	0.043
3	1	2:157737618	*ACVR1*	AT/G		loss of function	0.052
3	1	2:177617738	*TTC30A*	T/C		missense	0.035
3	1	2:178589758	*TTN*	G/A		missense	0.053
3	1	2:219567097	*OBSL1*	C/T		missense	0.052
3	1	2:30743428	*CAPN13*	G/C		missense	0.061
3	1	2:50466482	*NRXN1*	A/G		synonymous or non-coding sequence	0.053
3	1	2:98616297	*UNC50*	T/G		missense	0.054
3	1	20:43702538	*MYBL2*	C/T		missense	0.070
3	1	22:21661140	*intergenic*	C/A		synonymous or non-coding sequence	0.053
3	1	3:15707942	*ANKRD28,BTD*	G/A		missense	0.058
3	1	3:185689461	*IGF2BP2*	G/A		loss of function	0.056
3	1	3:193262978	*PLAAT1*	C/G		missense	0.074
3	1	3:47846797	*DHX30*	C/G		synonymous or non-coding sequence	0.042
3	1	3:62550073	*CADPS*	C/T		missense	0.048
3	1	4:1801421	*FGFR3*	C/T		missense	0.047
3	1	4:186271738	*F11*	C/T		missense	0.035
3	1	4:41613548	*LIMCH1*	C/G		missense	0.041
3	1	7:150237587	*ACTR3C*	G/A		synonymous or non-coding sequence	0.043
3	1	7:150742333	*GIMAP1-GIMAF*	C/T		missense	0.032
3	1	7:23165737	*KLHL7*	C/T		loss of function	0.090
3	1	7:45063977	*CCM2*	C/G		missense	0.055
3	1	7:99546959	*FAM200A*	T/C		synonymous or non-coding sequence	0.059
3	1	8:19960908	*LPL*	G/A		missense	0.043
3	1	8:2955701	*CSMD1*	G/A		synonymous or non-coding sequence	0.059
3	1	9:70344235	*SMC5*	G/A		missense	0.035
3	1	X:130230536	*ZNF280C*	G/T		loss of function	0.046
3	1	X:70420219	*KIF4A*	C/T		missense	0.083
4	1	12:4558868	*RAD51AP1*	G/C		missense	0.042
4	1	12:57204692	*LRP1*	G/A		missense	0.023
4	1	13:26046756	*SHISA2*	G/A		synonymous or non-coding sequence	0.035
4	1	20:38724793	*SLC32A1*	G/A		synonymous or non-coding sequence	0.043
4	1	22:25769230	*MYO18B*	A/G		synonymous or non-coding sequence	0.035
4	1	7:12333512	*VWDE*	A/T		missense	0.053
4	1	8:33389363	*FUT10*	A/C		missense	0.029
4	2	1:93877690	*DNTTIP2*	G/C		missense	0.043
4	2	16:11838057	*RSL1D1*	T/C		synonymous or non-coding sequence	0.042
4	2	16:19115553	*ITPRIPL2*	A/G		synonymous or non-coding sequence	0.036
4	2	17:80010306	*TBC1D16*	G/C		synonymous or non-coding sequence	0.032
4	2	20:38724793	*SLC32A1*	G/A		synonymous or non-coding sequence	0.038
4	2	21:41912362	*C2CD2*	G/A		missense	0.038
4	3	1:113929495	*HIPK1*	T/C		synonymous or non-coding sequence	0.039
4	3	1:197143629	*ASPM*	T/G		missense	0.049
4	3	1:202733702	*KDM5B*	C/T		missense	0.040
4	3	1:51311311	*TTC39A*	G/A		synonymous or non-coding sequence	0.036
4	3	10:119452729	*GRK5*	G/C		missense	0.086
4	3	10:28681458	*BAMBI*	A/G		missense	0.053
4	3	10:97185533	*SLIT1*	G/A		missense	0.044
4	3	13:111292223	*ARHGEF7*	C/T		missense	0.043
4	3	14:21352687	*SUPT16H*	T/C		missense	0.049
4	3	16:20987834	*DNAH3*	C/A		synonymous or non-coding sequence	0.069
4	3	16:74774604	*FA2H*	C/A		missense	0.061
4	3	19:43769541	*KCNN4*	C/T		missense	0.074
4	3	19:45064426	*CLASRP*	C/G		missense	0.055
4	3	19:54594463	*LILRA1*	C/A		synonymous or non-coding sequence	0.024
4	3	3:155775779	*C3orf33*	G/A		loss of function	0.050
4	3	4:67678512	*UBA6*	C/G		missense	0.045
4	3	5:124647309	*ZNF608*	C/T		synonymous or non-coding sequence	0.047
4	3	5:158712217	*EBF1*	T/C		missense	0.049
4	3	5:176868795	*UNC5A*	C/G		synonymous or non-coding sequence	0.041

4	3	6:133525307	*EYA4*	T/C	synonymous or non-coding sequence	0.045
4	3	7:150742414	*GIMAP1-GIMAF*	A/G	missense	0.034
4	3	8:143983592	*PARP10*	G/C	missense	0.040
4	3	9:137156865	*GRIN1*	A/G	missense	0.036
4	3	X:106205799	*PWWP3B*	C/T	loss of function	0.034
4	3	X:133217411	*TFDP3*	G/C	missense	0.010
4	4	12:57204692	*LRP1*	G/A	missense	0.028
4	4	13:26046756	*SHISA2*	G/A	synonymous or non-coding sequence	0.030
4	4	7:12333512	*VWDE*	A/T	missense	0.026
4	4	8:143983592	*PARP10*	G/C	missense	0.025
4	4	8:33389363	*FUT10*	A/C	missense	0.029
4	5	15:63599160	*FBXL22*	A/T	missense	0.059
4	5	16:11838057	*RSL1D1*	T/C	synonymous or non-coding sequence	0.062
4	5	17:7355225	*TMEM95*	C/T	synonymous or non-coding sequence	0.073
4	5	20:38724793	*SLC32A1*	G/A	synonymous or non-coding sequence	0.086
4	5	21:41912362	*C2CD2*	G/A	missense	0.058
4	5	5:141404856	*PCDHGA1,PCD*	G/T	missense	0.028
4	5	9:87886540	*SPATA31E1*	G/A	missense	0.060
5	1	13:61412430	*PCDH20*	G/T	missense	0.101
5	1	17:42852116	*AOC3*	G/A	missense	0.144
5	1	18:31469176	*DSG3*	G/A	missense	0.098
5	1	3:66417259	*LRIG1*	T/C	missense	0.124
5	1	9:87887768	*SPATA31E1*	C/T	missense	0.095
5	1	X:24211248	*ZFX*	C/T	missense	0.105
5	2	18:31338418	*DSG1*	G/A	missense	0.138
5	2	9:122568064	*OR1L8*	GCTCAT/-	missense	0.059
5	2	X:141908350	*MAGEC1*	C/T	synonymous or non-coding sequence	0.057
5	2	10:71279905	*UNC5B*	A/G	missense	0.055
5	2	17:10454790	*MYH4*	T/A	missense	0.070
5	2	4:442932	*ZNF721*	C/A	missense	0.060
5	2	X:154542444	*G6PD,IKBKG*	A/-	loss of function	0.077
5	2	X:80734167	*BRWD3*	T/C	missense	0.057
5	2	11:111974125	*DIXDC1*	C/T	missense	0.084
5	2	11:64903299	*ATG2A*	C/G	missense	0.053
5	2	12:101357071	*UTP20*	C/T	missense	0.096
5	2	12:297119	*KDM5A*	C/A	missense	0.042
5	2	16:29744510	*C16orf54*	G/T	synonymous or non-coding sequence	0.047
5	2	16:68255950	*PLA2G15*	C/T	synonymous or non-coding sequence	0.058
5	2	16:68907470	*TANGO6*	C/G	loss of function	0.088
5	2	16:75529639	*CHST5*	C/-	loss of function	0.093
5	2	17:10495019	*MYH1*	G/A	missense	0.047
5	2	17:58312305	*TSPOAP1*	G/A	missense	0.086
5	2	19:11832673	*ZNF440*	A/G	synonymous or non-coding sequence	0.080
5	2	19:48806664	*BCAT2*	G/A	synonymous or non-coding sequence	0.090
5	2	3:14898084	*FGD5*	C/T	missense	0.057
5	2	4:25676723	*SLC34A2*	TC/A	loss of function	0.055
5	2	5:141009720	*PCDHA1,PCDH*	C/T	missense	0.079
5	2	6:136038371	*PDE7B*	C/T	synonymous or non-coding sequence	0.050
5	2	6:137001598	*IL20RA*	AAT/-	missense	0.075
5	2	6:89664537	*MDN1*	A/C	missense	0.057
5	2	7:151049240	*ASIC3*	C/T	missense	0.041
5	2	7:151781157	*PRKAG2*	C/G	missense	0.071
5	2	7:36528331	*AOAH*	T/C	synonymous or non-coding sequence	0.080
5	2	8:54627068	*RP1*	G/A	synonymous or non-coding sequence	0.057
5	2	9:2186210	*SMARCA2*	G/A	missense	0.054
5	2	X:131285412	*IGSF1*	C/A	missense	0.038
5	2	X:141908350	*MAGEC1*	C/T	synonymous or non-coding sequence	0.013
5	2	X:14607140	*GLRA2*	C/T	missense	0.047
6	1	1:227733970	*JMJD4,SNAP47*	T/C	missense	0.045
6	1	1:90018692	*ZNF326*	T/C	missense	0.069
6	1	3:97874387	*CRYBG3*	A/C	missense	0.061
6	1	4:73139950	*ANKRD17*	A/C	missense	0.048
6	1	7:36357239	*KIAA0895*	C/A	missense	0.039
6	1	X:151181019	*GPR50*	G/C	missense	0.047
6	2	1:159931556	*IGSF9*	G/A	synonymous or non-coding sequence	0.030
6	2	1:197427632	*CRB1*	C/T	synonymous or non-coding sequence	0.031
6	2	1:227733970	*JMJD4,SNAP47*	T/C	missense	0.035
6	2	1:90018692	*ZNF326*	T/C	missense	0.049
6	2	12:18738375	*CAPZA3*	T/A	missense	0.033
6	2	16:3383109	*ZSCAN32*	T/G	synonymous or non-coding sequence	0.042
6	2	17:76882282	*MGAT5B*	C/T	missense	0.034
6	2	3:134371428	*AMOTL2*	C/T	synonymous or non-coding sequence	0.033
6	2	3:21860759	*intergenic*	T/C	synonymous or non-coding sequence	0.037
6	2	3:97874387	*CRYBG3*	A/C	missense	0.043

6	2	4:73139950	*ANKRD17*	A/C	missense	0.036
6	2	6:89701602	*MDN1*	T/C	missense	0.039
6	2	7:36357239	*KIAA0895*	C/A	missense	0.037
6	2	X:151181019	*GPR50*	G/C	missense	0.035
6	3	6:51659790	*PKHD1*	C/T	missense	0.063
6	3	X:123428115	*GRIA3*	C/T	synonymous or non-coding sequence	0.048
6	4	6:51659790	*PKHD1*	C/T	missense	0.054
6	4	X:123428115	*GRIA3*	C/T	synonymous or non-coding sequence	0.051
6	5	1:207475049	*CR2*	C/A	missense	0.110
6	5	1:29260828	*PTPRU*	G/A	missense	0.026
6	5	1:88833348	*PKN2*	C/G	loss of function	0.074
6	5	12:82476714	*METTL25*	A/G	missense	0.071
6	5	15:43432275	*TP53BP1*	A/C	missense	0.132
6	5	17:10301687	*MYH13*	G/A	missense	0.112
6	5	19:38412799	*RASGRP4*	CTG/-	missense	0.094
6	5	19:42370749	*MEGF8*	G/A	missense	0.124
6	5	19:58416906	*ZNF584*	C/T	missense	0.109
6	5	2:184866380	*ZNF804A*	G/C	missense	0.080
6	5	3:184230793	*VWA5B2*	C/G	missense	0.092
6	5	3:38190773	*OXSR1*	G/T	missense	0.090
6	5	6:127330931	*ECHDC1*	T/C	missense	0.091
6	5	6:127515824	*SOGA3*	-/A	loss of function	0.103
6	5	X:153866732	*L1CAM*	G/T	missense	0.025
6	5	X:66028103	*VSIG4*	T/A	missense	0.080
6	6	6:51659790	*PKHD1*	C/T	missense	0.041
6	6	X:123428115	*GRIA3*	C/T	synonymous or non-coding sequence	0.030
6	7	X:123428115	*GRIA3*	C/T	synonymous or non-coding sequence	0.049
6	8	1:207475049	*CR2*	C/A	missense	0.028
6	8	1:88833348	*PKN2*	C/G	loss of function	0.053
6	8	15:43432275	*TP53BP1*	A/C	missense	0.035
6	8	15:59521327	*FAM81A*	C/T	synonymous or non-coding sequence	0.033
6	8	17:10301687	*MYH13*	G/A	missense	0.044
6	8	19:38412799	*RASGRP4*	CTG/-	missense	0.034
6	8	19:42370749	*MEGF8*	G/A	missense	0.056
6	8	19:58416906	*ZNF584*	C/T	missense	0.032
6	8	20:20191394	*CFAP61*	G/T	missense	0.056
6	8	3:184230793	*VWA5B2*	C/G	missense	0.030
6	8	6:127330931	*ECHDC1*	T/C	missense	0.057
6	8	X:66028103	*VSIG4*	T/A	missense	0.042
6	9	X:123428115	*GRIA3*	C/T	synonymous or non-coding sequence	0.050
6	9	X:48602179	*WDR13*	C/T	missense	0.034
6	10	X:48602179	*WDR13*	C/T	missense	0.026
7	1	19:12705249	*TNPO2*	C/T	synonymous or non-coding sequence	0.102
7	2	1:169607125	*SELP*	C/T	missense	0.062
7	2	1:182057377	*ZNF648*	C/A	missense	0.066
7	2	10:5641843	*ASB13*	G/A	synonymous or non-coding sequence	0.029
7	2	11:112193664	*BCO2*	A/G	missense	0.052
7	2	11:124894007	*ROBO4*	G/A	missense	0.067
7	2	12:54399733	*ITGA5*	CTG/-	missense	0.043
7	2	14:34776440	*BAZ1A*	G/C	missense	0.100
7	2	15:78101233	*SH2D7*	C/T	missense	0.085
7	2	15:82681537	*AP3B2*	G/C	missense	0.031
7	2	17:82093742	*FASN*	G/T	missense	0.056
7	2	18:78994657	*SALL3*	G/A	missense	0.064
7	2	19:39489817	*TIMM50*	C/G	synonymous or non-coding sequence	0.044
7	2	19:56027291	*NLRP5*	G/A	missense	0.037
7	2	19:8089911	*FBN3*	G/A	missense	0.044
7	2	2:156330026	*NR4A2*	G/A	missense	0.054
7	2	2:208330554	*PIKFYVE*	G/T	missense	0.034
7	2	2:219023220	*CFAP65*	G/T	missense	0.056
7	2	21:44119403	*PWP2*	G/C	synonymous or non-coding sequence	0.039
7	2	22:41732567	*MEI1*	C/T	missense	0.050
7	2	3:122261683	*CASR*	C/T	synonymous or non-coding sequence	0.044
7	2	4:154612139	*FGG*	T/C	synonymous or non-coding sequence	0.074
7	2	4:947808	*TMEM175*	C/T	missense	0.043
7	2	6:138334768	*ARFGEF3*	C/T	synonymous or non-coding sequence	0.063
7	2	6:70857974	*B3GAT2,SMAP*	A/G	synonymous or non-coding sequence	0.059
7	2	6:73224327	*KHDC1L*	C/T	missense	0.058
7	2	7:130400184	*CEP41*	C/A	missense	0.078
7	2	8:1549530	*DLGAP2*	G/C	synonymous or non-coding sequence	0.043
7	2	8:74361953	*GDAP1*	A/G	missense	0.047
7	2	9:133102804	*RALGDS*	C/T	missense	0.063
7	2	X:154360190	*FLNA*	C/A	missense	0.028
7	2	X:73447544	*CDX4*	G/A	synonymous or non-coding sequence	0.019

7	3	19:12705249	*TNPO2*	C/T	synonymous or non-coding sequence	0.032
8	1	1:152032760	*S100A11*	G/A	loss of function	0.027
8	1	10:113141246	*TCF7L2*	G/A	synonymous or non-coding sequence	0.038
8	1	10:48206921	*FRMPD2*	G/C	missense	0.041
8	1	11:66347337	*B4GAT1*	C/-	loss of function	0.031
8	1	15:45117865	*DUOXA1,DUOX*	C/T	loss of function	0.033
8	1	16:1220384	*CACNA1H*	A/G	missense	0.060
8	1	16:29968140	*TMEM219*	C/T	synonymous or non-coding sequence	0.044
8	1	16:57432507	*CIAPIN1*	C/T	missense	0.047
8	1	16:58280453	*CCDC113,PRS*	A/G	missense	0.051
8	1	2:27843073	*RBKS*	T/-	loss of function	0.033
8	1	2:88885846	*intergenic*	A/C	synonymous or non-coding sequence	0.038
8	1	22:22881220	*intergenic*	A/G	synonymous or non-coding sequence	0.044
8	1	4:118318414	*PRSS12*	T/G	missense	0.051
8	1	5:13777208	*DNAH5*	T/A	synonymous or non-coding sequence	0.042
8	1	6:117317230	*ROS1*	A/G	synonymous or non-coding sequence	0.052
8	1	6:26250294	*H3C7*	G/C	synonymous or non-coding sequence	0.065
8	1	7:117792335	*CTTNBP2*	A/G	synonymous or non-coding sequence	0.053
8	1	7:124746612	*GPR37*	G/A	synonymous or non-coding sequence	0.058
8	1	9:21217198	*IFNA16*	C/T	synonymous or non-coding sequence	0.020
8	1	X:12702004	*FRMPD4*	A/G	missense	0.033
8	1	X:12720735	*FRMPD4*	G/A	missense	0.025
8	2	1:152032760	*S100A11*	G/A	loss of function	0.039
8	2	1:43603783	*PTPRF*	C/G	missense	0.036
8	2	10:113141246	*TCF7L2*	G/A	synonymous or non-coding sequence	0.028
8	2	11:4989690	*MMP26*	C/T	missense	0.034
8	2	11:67279636	*GRK2*	A/T	missense	0.044
8	2	11:67279637	*GRK2*	G/T	missense	0.044
8	2	12:48837534	*DDX23*	T/C	missense	0.045
8	2	15:24676062	*NPAP1*	C/T	synonymous or non-coding sequence	0.050
8	2	15:45117865	*DUOXA1,DUOX*	C/T	loss of function	0.035
8	2	16:58280453	*CCDC113,PRS*	A/G	missense	0.047
8	2	6:117317230	*ROS1*	A/G	synonymous or non-coding sequence	0.030
8	2	6:26250294	*H3C7*	G/C	synonymous or non-coding sequence	0.053
8	2	7:124746612	*GPR37*	G/A	synonymous or non-coding sequence	0.043
8	2	8:51411364	*PXDNL*	G/A	missense	0.047
8	2	9:21217198	*IFNA16*	C/T	synonymous or non-coding sequence	0.032
8	2	X:154439134	*GDI1*	G/C	missense	0.039
8	2	16:57432507	*CIAPIN1*	C/T	missense	0.021
8	3	16:58280453	*CCDC113,PRS*	A/G	missense	0.043
8	3	6:26250294	*H3C7*	G/C	synonymous or non-coding sequence	0.028
8	3	7:117792335	*CTTNBP2*	A/G	synonymous or non-coding sequence	0.032
9	1	1:201224867	*IGFN1*	G/T	missense	0.059
9	1	16:68183283	*NFATC3*	T/-	loss of function	0.059
9	1	3:120644421	*HGD*	A/T	synonymous or non-coding sequence	0.078
9	1	4:97840819	*STPG2*	A/-	loss of function	0.059
9	1	8:94491850	*VIRMA*	C/T	missense	0.047
9	1	X:54132818	*FAM120C*	C/T	missense	0.035
9	1	X:68715365	*STARD8*	G/A	missense	0.049
9	2	15:40220573	*BUB1B,BUB1B-*	T/C	synonymous or non-coding sequence	0.110
9	2	17:41440511	*KRT38*	G/A	synonymous or non-coding sequence	0.036
9	2	19:36908409	*ZNF829*	C/T	loss of function	0.060
9	2	19:5896574	*NDUFA11*	A/C	synonymous or non-coding sequence	0.085
9	2	2:130763072	*AMER3*	G/C	missense	0.045
9	2	20:10055628	*ANKEF1*	G/T	missense	0.082
9	2	3:38728561	*SCN10A*	A/G	missense	0.038
9	2	7:20785032	*SP8*	A/G	missense	0.083
9	2	X:131058430	*ARHGAP36*	C/T	missense	0.131
10	1	5:81344975	*ACOT12*	G/A	synonymous or non-coding sequence	0.081
10	1	9:110800738	*MUSK*	C/T	missense	0.099
10	3	17:28579209	*SPAG5*	G/A	loss of function	0.033
10	3	6:160073328	*IGF2R*	C/T	synonymous or non-coding sequence	0.041
10	3	X:50194368	*AKAP4*	G/A	synonymous or non-coding sequence	0.040
11	1	17:75892489	*TRIM65*	G/A	synonymous or non-coding sequence	0.059
11	1	18:79863868	*KCNG2*	C/G	missense	0.053
11	2	1:159928785	*IGSF9*	C/T	missense	0.071
11	2	12:106070842	*NUAK1*	C/A	missense	0.052
11	2	18:79863868	*KCNG2*	C/G	missense	0.110
12	1	1:209782927	*C1orf74*	G/A	synonymous or non-coding sequence	0.114
12	1	12:56164428	*SMARCC2*	G/A	missense	0.098
12	2	1:209782927	*C1orf74*	G/A	synonymous or non-coding sequence	0.062
12	2	12:56164428	*SMARCC2*	G/A	missense	0.044
12	3	12:121888863	*PSMD9*	G/A	missense	0.048

Supplemental Table S4

gene	mim_morbid_accession	mim_morbid_description
A2M	NA	
ABCA7	608907	ALZHEIMER DISEASE 9, SUSCEPTIBILITY TO; AD9;;ALZHEIMER DISEASE 9, LATE-ONSET
ACOT12	NA	
ACOXL	NA	
ACP7	NA	
ACTR3C	NA	
ACVR1	135100	FIBRODYSPLASIA OSSIFICANS PROGRESSIVA; FOP
ADAM7	NA	
ADAMTS1	NA	
ADCY8	NA	
ADGRV1	604352	FEBRILE SEIZURES, FAMILIAL, 4; FEB4;;CONVULSIONS, FAMILIAL FEBRILE, 4
ADGRV1	605472	USHER SYNDROME, TYPE IIC; USH2C USHER SYNDROME, TYPE IIC, GPR98/PDZD7, DIGENIC, INCLUDED;;USHER SYNDROME, TYPE IIB, FORMERLY, INCLUDED; USH2B, FORMERLY, INCLUDED
AKAP4	NA	
AKR1C4	614279	46,XY SEX REVERSAL 8; SRXY8;;MALE PSEUDOHERMAPHRODITISM DUE TO DEFICIENCY OF TESTICULAR 17,20-DESMOLASE; TDD
ALG13	300884	DEVELOPMENTAL AND EPILEPTIC ENCEPHALOPATHY 36; DEE36;;EPILEPTIC ENCEPHALOPATHY, EARLY INFANTILE, 36; EIEE36;;CONGENITAL DISORDER OF GLYCOSYLATION, TYPE Is; CDG1S;;CDG Is; CDGIs
AMER3	NA	
AMOTL2	NA	
ANKEF1	NA	
ANKRD17	619504	CHOPRA-AMIEL-GORDON SYNDROME; CAGS
ANKRD28	NA	
ANKRD33	NA	
AOAH	NA	
AOC3	NA	
AP3B2	617276	DEVELOPMENTAL AND EPILEPTIC ENCEPHALOPATHY 48; DEE48;;EPILEPTIC ENCEPHALOPATHY, EARLY INFANTILE, 48; EIEE48
ARFGEF3	NA	
ARHGAP36	NA	
ARHGDIA	615244	NEPHROTIC SYNDROME, TYPE 8; NPHS8
ARHGEF7	NA	
ARMH1	NA	
ASB13	NA	
ASIC3	NA	
ASPM	608716	MICROCEPHALY 5, PRIMARY, AUTOSOMAL RECESSIVE; MCPH5
ATE1	NA	
ATG2A	NA	
ATP10B	NA	
ATP6V0A4	602722	RENAL TUBULAR ACIDOSIS, DISTAL, 3, WITH OR WITHOUT SENSORINEURAL HEARING LOSS; DRTA3;;RTADR
ATP9B	NA	
B3GALT5	NA	
B3GAT2	NA	
B4GAT1	615287	MUSCULAR DYSTROPHY-DYSTROGLYCANOPATHY (CONGENITAL WITH BRAIN AND EYE ANOMALIES), TYPE A, 13; MDDGA13;;WALKER-WARBURG SYNDROME OR MUSCLE-EYE-BRAIN DISEASE, B3GNT1-RELATED
BAMBI	NA	
BAZ1A	NA	
BCAT2	618850	HYPERVALINEMIA AND HYPERLEUCINE-ISOLEUCINEMIA; HVLI;;BRANCHED-CHAIN AMINOTRANSFERASE DEFICIENCY
BCO2	NA	
BCOR	300166	MICROPHTHALMIA, SYNDROMIC 2; MCOPS2;;OCULOFACIOCARDIODENTAL SYNDROME;;OFCD SYNDROME;;MICROPHTHALMIA, CATARACTS, RADICULOMEGALY, AND SEPTAL HEART DEFECTS;;ANOP2, FORMERLY;;MAA2, FORMERLY
BCOR	309800	MICROPHTHALMIA, SYNDROMIC 1; MCOPS1;;LENZ MICROPHTHALMIA SYNDROME;;LENZ DYSPLASIA;;MICROPHTHALMIA, SYNDROMIC 4, FORMERLY; MCOPS4, FORMERLY;;ANOP1, FORMERLY;;MAA, FORMERLY
BPIFC	NA	
BRWD3	300659	INTELLECTUAL DEVELOPMENTAL DISORDER, X-LINKED 93; XLID93;;MENTAL RETARDATION, X-LINKED 93; MRX93;;MENTAL RETARDATION, X-LINKED, WITH MACROCEPHALY
BTD	253260	BIOTINIDASE DEFICIENCY;;BTD DEFICIENCY;;MULTIPLE CARBOXYLASE DEFICIENCY, LATE-ONSET;;MULTIPLE CARBOXYLASE DEFICIENCY, JUVENILE-ONSET
BUB1B	114500	COLORECTAL CANCER; CRC;;COLON CANCER
BUB1B	176430	PREMATURE CHROMATID SEPARATION TRAIT; PCS;;TOTAL PREMATURE CHROMATID SEPARATION TRAIT
BUB1B	257300	MOSAIC VARIEGATED ANEUPLOIDY SYNDROME 1; MVA1;;MVA SYNDROME
BUB1B-PAK6	NA	
C10orf95	NA	
C16orf54	NA	
C1orf74	NA	
C2	217000	COMPLEMENT COMPONENT 2 DEFICIENCY; C2D;;C2 DEFICIENCY
C2	615489	MACULAR DEGENERATION, AGE-RELATED, 14; ARMD14 MACULAR DEGENERATION, AGE-RELATED, REDUCED RISK OF, INCLUDED
C2CD2	NA	
C3orf33	NA	

C9orf57	NA	
CACNA1C	601005	TIMOTHY SYNDROME; TS;;LONG QT SYNDROME WITH SYNDACTYLY
CACNA1C	611875	BRUGADA SYNDROME 3; BRGDA3
CACNA1C	618447	LONG QT SYNDROME 8; LQT8
CACNA1C	620029	NEURODEVELOPMENTAL DISORDER WITH HYPOTONIA, LANGUAGE DELAY, AND SKELETAL DEFECTS WITH OR WITHOUT SEIZURES; NEDHLSS
CACNA1H	611942	EPILEPSY, CHILDHOOD ABSENCE, SUSCEPTIBILITY TO, 6; ECA6 EPILEPSY, IDIOPATHIC GENERALIZED, SUSCEPTIBILITY TO, 6, INCLUDED; EIG6, INCLUDED
CACNA1H	617027	HYPERALDOSTERONISM, FAMILIAL, TYPE IV; HALD4;;FH IV;;ALDOSTERONISM, PRIMARY, AND HYPERTENSION
CADPS	NA	
CAMKV	NA	
CAPN13	NA	
CAPZA3	NA	
CASR	145980	HYPOCALCIURIC HYPERCALCEMIA, FAMILIAL, TYPE I; HHC1;;HHC; FHH;;FHH1;;FAMILIAL BENIGN HYPERCALCEMIA 1; FBH1;;HYPERCALCEMIA, FAMILIAL BENIGN HYPOCALCIURIC HYPERCALCEMIA, ACQUIRED, INCLUDED
CASR	239200	HYPERPARATHYROIDISM, NEONATAL SEVERE; NSHPT;;NSPH; NHPT;;HYPERPARATHYROIDISM, NEONATAL SEVERE PRIMARY
CASR	601198	HYPOCALCEMIA, AUTOSOMAL DOMINANT 1; HYPOC1;;HYPERCALCIURIC HYPOCALCEMIA;;HYPOCALCEMIA, FAMILIAL HYPOCALCEMIA, AUTOSOMAL DOMINANT 1, WITH BARTTER SYNDROME, INCLUDED
CASR	612899	EPILEPSY, IDIOPATHIC GENERALIZED, SUSCEPTIBILITY TO, 8; EIG8
CCDC113	NA	
CCM2	603284	CEREBRAL CAVERNOUS MALFORMATIONS 2; CCM2
CCNA1	NA	
CD247	610163	IMMUNODEFICIENCY 25; IMD25;;IMMUNODEFICIENCY DUE TO DEFECT IN CD3-ZETA
CDC14B	NA	
CDX4	NA	
CEP41	614464	JOUBERT SYNDROME 15; JBTS15 JOUBERT SYNDROME 9/15, DIGENIC, INCLUDED;;JOUBERT SYNDROME 12/15, DIGENIC, INCLUDED
CEP70	NA	
CFAP61	620409	SPERMATOGENIC FAILURE 84; SPGF84
CFAP65	618664	SPERMATOGENIC FAILURE 40; SPGF40
CFI	610984	COMPLEMENT FACTOR I DEFICIENCY; CFID;;C3 GLOMERULOPATHY 2; C3G2;;COMPLEMENT COMPONENT 3 INACTIVATOR DEFICIENCY;;C3 INACTIVATOR DEFICIENCY
CFI	612923	HEMOLYTIC UREMIC SYNDROME, ATYPICAL, SUSCEPTIBILITY TO, 3; AHUS3;;AHUS, SUSCEPTIBILITY TO, 3
CFI	615439	MACULAR DEGENERATION, AGE-RELATED, 13; ARMD13
CHD3	618205	SNIJDERS BLOK-CAMPEAU SYNDROME; SNIBCPS;;INTELLECTUAL DEVELOPMENTAL DISORDER WITH MACROCEPHALY, SPEECH DELAY, AND DYSMORPHIC FACIES; IDDMSF
CHD7	214800	CHARGE SYNDROME;;CHARGE ASSOCIATION--COLOBOMA, HEART ANOMALY, CHOANAL ATRESIA, RETARDATION, GENITAL AND EAR ANOMALIES;;HALL-HITTNER SYNDROME; HHS
CHD7	612370	HYPOGONADOTROPIC HYPOGONADISM 5 WITH OR WITHOUT ANOSMIA; HH5
CHRNA4	188890	TOBACCO ADDICTION, SUSCEPTIBILITY TO;;CIGARETTE HABITUATION, SUSCEPTIBILITY TO;;SMOKING HABIT, SUSCEPTIBILITY TO;;NICOTINE DEPENDENCE, SUSCEPTIBILITY TO;;NICOTINE ADDICTION, SUSCEPTIBILITY TO;;NICOTINE DEPENDENCE, PROTECTION AGAINST
CHRNA4	600513	EPILEPSY, NOCTURNAL FRONTAL LOBE, 1; ENFL1
CHST5	NA	
CHST9	NA	
CIAPIN1	NA	
CLASRP	NA	
CLRN2	619174	DEAFNESS, AUTOSOMAL RECESSIVE 117; DFNB117
CLSTN2	NA	
CNTN5	NA	
CNTNAP4	NA	
COL4A3	104200	ALPORT SYNDROME 3, AUTOSOMAL DOMINANT; ATS3
COL4A3	203780	ALPORT SYNDROME 2, AUTOSOMAL RECESSIVE; ATS2
COL4A3	620320	HEMATURIA, BENIGN FAMILIAL, 2; BFH2
CPA2	NA	
CPSF2	NA	
CR2	240500	IMMUNODEFICIENCY, COMMON VARIABLE, 2; CVID2;;ANTIBODY DEFICIENCY DUE TO TACI DEFECT;;HYPOGAMMAGLOBULINEMIA DUE TO TACI DEFICIENCY
CR2	610927	SYSTEMIC LUPUS ERYTHEMATOSUS, SUSCEPTIBILITY TO, 9; SLEB9
CR2	614699	IMMUNODEFICIENCY, COMMON VARIABLE, 7; CVID7
CRB1	172870	PIGMENTED PARAVENOUS CHORIORETINAL ATROPHY; PPCRA
CRB1	600105	RETINITIS PIGMENTOSA 12; RP12;;RETINITIS PIGMENTOSA WITH OR WITHOUT PARAARTERIOLAR PRESERVATION OF RETINAL PIGMENT EPITHELIUM;;RP WITH OR WITHOUT PRESERVED PARAARTERIOLE RETINAL PIGMENT EPITHELIUM;;RP WITH OR WITHOUT PPRPE
CRB1	613835	LEBER CONGENITAL AMAUROSIS 8; LCA8
CRYBG3	NA	
CSMD1	NA	
CTTNBP2	NA	
CWH43	NA	
CXXC4	NA	
CYC1	615453	MITOCHONDRIAL COMPLEX III DEFICIENCY, NUCLEAR TYPE 6; MC3DN6

CYP2B6	614546	EFAVIRENZ, POOR METABOLISM OF EFAVIRENZ CENTRAL NERVOUS SYSTEM TOXICITY, SUSCEPTIBILITY TO, INCLUDED
CYP4B1	NA	
DAGLA	NA	
DDAH2	NA	
DDX23	NA	
DGKA	NA	
DHX30	617804	NEURODEVELOPMENTAL DISORDER WITH VARIABLE MOTOR AND LANGUAGE IMPAIRMENT; NEDMIAL
DIXDC1	NA	
DLGAP2	NA	
DNAH3	NA	
DNAH5	608644	CILIARY DYSKINESIA, PRIMARY, 3; CILD3;;CILIARY DYSKINESIA, PRIMARY, 3, WITH OR WITHOUT SITUS INVERSUS
DNAI3	NA	
DNAJB4	620326	CONGENITAL MYOPATHY 21 WITH EARLY RESPIRATORY FAILURE; CMYP21
DNTTIP2	NA	
DPY19L2	613958	SPERMATOGENIC FAILURE 9; SPGF9;;GLOBOZOOSPERMIA, COMPLETE;;GLOBOZOOSPERMIA, TOTAL
DSG1	148700	PALMOPLANTAR KERATODERMA I, STRIATE, FOCAL, OR DIFFUSE; PPKS1;;KERATOSIS PALMOPLANTARIS STRIATA I;;STRIATE PALMOPLANTAR KERATODERMA I; SPPK1;;KERATODERMA, PALMOPLANTAR, STRIATE FORM I; KPPS1
DSG1	615508	ERYTHRODERMA, CONGENITAL, WITH PALMOPLANTAR KERATODERMA, HYPOTRICHOSIS, AND HYPER-IgE; EPKHE;;SEVERE DERMATITIS, MULTIPLE ALLERGIES, AND METABOLIC WASTING SYNDROME;;SAM SYNDROME
DSG3	619226	BLISTERING, ACANTHOLYTIC, OF ORAL AND LARYNGEAL MUCOSA; ABOLM
DUOX1	NA	
DUOXA1	NA	
DUOXA2	274900	THYROID DYSHORMONOGENESIS 5; TDH5;;THYROID HORMONOGENESIS, GENETIC DEFECT IN, 5;;HYPOTHYROIDISM, CONGENITAL, DUE TO DYSHORMONOGENESIS, 5
DVL1	180700	ROBINOW SYNDROME, AUTOSOMAL DOMINANT 1; DRS1;;ROBINOW DWARFISM;;FETAL FACE SYNDROME;;ACRAL DYSOSTOSIS WITH FACIAL AND GENITAL ABNORMALITIES
DVL1	616331	ROBINOW SYNDROME, AUTOSOMAL DOMINANT 2; DRS2
EBF1	NA	
ECHDC1	NA	
EFNA1	NA	
EFTUD2	610536	MANDIBULOFACIAL DYSOSTOSIS, GUION-ALMEIDA TYPE; MFDGA;;MANDIBULOFACIAL DYSOSTOSIS WITH MICROCEPHALY; MFDM;;GROWTH AND MENTAL RETARDATION, MANDIBULOFACIAL DYSOSTOSIS, MICROCEPHALY, AND CLEFT PALATE
ELF4	301074	AUTOINFLAMMATORY SYNDROME, FAMILIAL, X-LINKED, BEHCET-LIKE 2; AIFBL2;;DEFICIENCY IN ELF4, X-LINKED; DEX
EPPK1	NA	
EPS15L1	NA	
ERICH1	NA	
EVPL	NA	
EXOSC4	NA	
EYA4	601316	DEAFNESS, AUTOSOMAL DOMINANT 10; DFNA10
EYA4	605362	CARDIOMYOPATHY, DILATED, 1J; CMD1J;;CARDIOMYOPATHY, DILATED, WITH SENSORINEURAL HEARING LOSS, AUTOSOMAL DOMINANT
F11	612416	FACTOR XI DEFICIENCY;;F11 DEFICIENCY;;PLASMA THROMBOPLASTIN ANTECEDENT DEFICIENCY;;PTA DEFICIENCY;;ROSENTHAL SYNDROME
F5	188055	THROMBOPHILIA DUE TO ACTIVATED PROTEIN C RESISTANCE; THPH2;;ACTIVATED PROTEIN C RESISTANCE;;APC RESISTANCE;;THROMBOPHILIA DUE TO DEFICIENCY OF ACTIVATED PROTEIN C COFACTOR;;PROC COFACTOR DEFICIENCY;;PCCF DEFICIENCY;;THROMBOPHILIA V THROMBOPHILIA DUE /.../CTOR V LEIDEN, INCLUDED
F5	227400	FACTOR V DEFICIENCY;;PARAHEMOPHILIA;;OWREN PARAHEMOPHILIA;;LABILE FACTOR DEFICIENCY
F5	600880	BUDD-CHIARI SYNDROME; BDCHS MEMBRANOUS OBSTRUCTION OF INFERIOR VENA CAVA, INCLUDED; MOVC, INCLUDED
F5	601367	STROKE, ISCHEMIC;;CEREBROVASCULAR ACCIDENT;;CEREBRAL INFARCTION
F5	614389	PREGNANCY LOSS, RECURRENT, SUSCEPTIBILITY TO, 1; RPRGL1;;RPRGL;;RPL;;ABORTION, SPONTANEOUS, RECURRENT;;FETAL LOSS, RECURRENT, SUSCEPTIBILITY TO;;MISCARRIAGE, RECURRENT;;EMBRYONIC LOSS, RECURRENT;;STILLBIRTH, RECURRENT
FA2H	612319	SPASTIC PARAPLEGIA 35, AUTOSOMAL RECESSIVE, WITH OR WITHOUT NEURODEGENERATION; SPG35;;FATTY ACID HYDROXYLASE-ASSOCIATED NEURODEGENERATION; FAHN;;LEUKODYSTROPHY, DYSMYELINATING, AND SPASTIC PARAPARESIS WITH OR WITHOUT DYSTONIA
FAM120C	NA	
FAM178B	NA	
FAM193B	NA	
FAM200A	NA	
FAM216A	NA	
FAM81A	NA	
FAM90A1	NA	
FASN	NA	
FBN3	NA	
FBXL22	NA	
FCHO1	619164	IMMUNODEFICIENCY 76; IMD76
FGA	105200	AMYLOIDOSIS, FAMILIAL VISCERAL;;AMYLOIDOSIS VIII;;OSTERTAG TYPE AMYLOIDOSIS;;GERMAN TYPE AMYLOIDOSIS;;AMYLOIDOSIS, FAMILIAL RENAL;;AMYLOIDOSIS, SYSTEMIC NONNEUROPATHIC
FGA	202400	AFIBRINOGENEMIA, CONGENITAL HYPOFIBRINOGENEMIA, CONGENITAL, INCLUDED

FGA	616004	DYSFIBRINOGENEMIA, CONGENITAL HYPODYSFIBRINOGENEMIA, CONGENITAL, INCLUDED
FGD5	NA	
FGFR3	100800	ACHONDROPLASIA; ACH
FGFR3	109800	BLADDER CANCER
FGFR3	114500	COLORECTAL CANCER; CRC;;COLON CANCER
FGFR3	146000	HYPOCHONDROPLASIA; HCH
FGFR3	162900	NEVUS, EPIDERMAL;;NEVUS, KERATINOCYTIC, NONEPIDERMOLYTIC NEVUS SEBACEOUS, INCLUDED;;NEVUS, WOOLLY HAIR, INCLUDED
FGFR3	187600	THANATOPHORIC DYSPLASIA, TYPE I; TD1;;THANATOPHORIC DYSPLASIA; TD;;THANATOPHORIC DWARFISM;;PLATYSPONDYLIC LETHAL SKELETAL DYSPLASIA, SAN DIEGO TYPE;;LETHAL SHORT-LIMBED PLATYSPONDYLIC DWARFISM, SAN DIEGO TYPE
FGFR3	187601	THANATOPHORIC DYSPLASIA, TYPE II; TD2;;THANATOPHORIC DYSPLASIA WITH STRAIGHT FEMURS AND CLOVERLEAF SKULL;;THANATOPHORIC DYSPLASIA WITH KLEEBLATTSCHAEDEL;;CLOVERLEAF SKULL WITH THANATOPHORIC DWARFISM
FGFR3	273300	TESTICULAR GERM CELL TUMOR; TGCT;;MALE GERM CELL TUMOR; MGCT SEMINOMA, INCLUDED;;NONSEMINOMATOUS GERM CELL TUMORS, INCLUDED;;TERATOMA, TESTICULAR, INCLUDED;;EMBRYONAL CELL CARCINOMA, INCLUDED;;ENDODERMAL SINUS TUMOR, INCLUDED;;SPERMATOCYTIC SEMINOMA /.../UDED
FGFR3	602849	MUENKE SYNDROME; MNKES;;MUENKE NONSYNDROMIC CORONAL CRANIOSYNOSTOSIS
FGFR3	603956	CERVICAL CANCER
FGFR3	610474	CAMPTODACTYLY, TALL STATURE, AND HEARING LOSS SYNDROME; CATSHLS;;CATSHL SYNDROME
FGFR3	612247	CROUZON SYNDROME WITH ACANTHOSIS NIGRICANS; CAN;;CROUZONODERMOSKELETAL SYNDROME
FGFR3	616482	ACHONDROPLASIA, SEVERE, WITH DEVELOPMENTAL DELAY AND ACANTHOSIS NIGRICANS; SADDAN;;SADDAN DYSPLASIA
FGFR3	620192	LACRIMOAURICULODENTODIGITAL SYNDROME 2; LADD2;;LADD SYNDROME 2
FGG	202400	AFIBRINOGENEMIA, CONGENITAL HYPOFIBRINOGENEMIA, CONGENITAL, INCLUDED
FGG	616004	DYSFIBRINOGENEMIA, CONGENITAL HYPODYSFIBRINOGENEMIA, CONGENITAL, INCLUDED
FKBP8	NA	
FLNA	300048	INTESTINAL PSEUDOOBSTRUCTION, NEURONAL, CHRONIC IDIOPATHIC, X-LINKED;;IPOX;;CONGENITAL IDIOPATHIC INTESTINAL PSEUDOOBSTRUCTION; CIIP;;CIIP, X-LINKED; CIIPX;;INTESTINAL PSEUDOOBSTRUCTION, NEURONAL, CHRONIC IDIOPATHIC, WITH CENTRAL NERVOUS SYSTEM INVO /.../T CONGENITAL SHORT BOWEL SYNDROME, X-LINKED, INCLUDED
FLNA	300049	PERIVENTRICULAR NODULAR HETEROTOPIA 1; PVNH1;;HETEROTOPIA, PERIVENTRICULAR, X-LINKED DOMINANT;;HETEROTOPIA, FAMILIAL NODULAR;;NODULAR HETEROTOPIA, BILATERAL PERIVENTRICULAR; NHBP; BPNH;;HETEROTOPIA, PERIVENTRICULAR, EHLERS-DANLOS VARIANT;;PERIVENTRI /.../NODULAR HETEROTOPIA 4, FORMERLY; PVNH4, FORMERLY HETEROTOPIA, PERIVENTRICULAR NODULAR, WITH FRONTOMETAPHYSEAL DYSPLASIA, INCLUDED
FLNA	300244	TERMINAL OSSEOUS DYSPLASIA; TOD;;TERMINAL OSSEOUS DYSPLASIA AND PIGMENTARY DEFECTS; TODPD;;ODPD;;OSSEOUS DYSPLASIA, DIGITAL, WITH FACIAL PIGMENTARY DEFECTS AND MULTIPLE FRENULA; ODPF;;ODPF SYNDROME
FLNA	300321	FG SYNDROME 2; FGS2
FLNA	304120	OTOPALATODIGITAL SYNDROME, TYPE II; OPD2;;OPD II SYNDROME;;OPD SYNDROME 2;;CRANIOORODIGITAL SYNDROME;;FACIOPALATOOSSEOUS SYNDROME; FPO
FLNA	305620	FRONTOMETAPHYSEAL DYSPLASIA 1; FMD1;;FMD
FLNA	309350	MELNICK-NEEDLES SYNDROME; MNS;;MELNICK-NEEDLES OSTEODYSPLASTY;;OSTEODYSPLASTY OF MELNICK AND NEEDLES
FLNA	311300	OTOPALATODIGITAL SYNDROME, TYPE I; OPD1;;OPD I SYNDROME;;OPD SYNDROME 1 OTOPALATODIGITAL SPECTRUM DISORDER, INCLUDED;;FRONTOOTOPALATODIGITAL OSTEODYSPLASIA, INCLUDED
FLNA	314400	CARDIAC VALVULAR DYSPLASIA, X-LINKED; CVDPX;;VALVULAR HEART DISEASE, CONGENITAL;;MYXOMATOUS VALVULAR DYSTROPHY, X-LINKED; XMVD;;EHLERS-DANLOS SYNDROME, TYPE V, FORMERLY; EDS5, FORMERLY
FNDC1	NA	
FPR2	NA	
FRMPD2	NA	
FRMPD4	300983	INTELLECTUAL DEVELOPMENTAL DISORDER, X-LINKED 104; XLID104;;MENTAL RETARDATION, X-LINKED 104; MRX104
FUT10	NA	
G6PD	300908	ANEMIA, NONSPHEROCYTIC HEMOLYTIC, DUE TO G6PD DEFICIENCY;;FAVISM, SUSCEPTIBILITY TO
G6PD	611162	MALARIA, SUSCEPTIBILITY TO MALARIA, RESISTANCE TO, INCLUDED;;MALARIA, SEVERE, SUSCEPTIBILITY TO, INCLUDED;;MALARIA, SEVERE, RESISTANCE TO, INCLUDED;;MALARIA, CEREBRAL, SUSCEPTIBILITY TO, INCLUDED;;MALARIA, CEREBRAL, RESISTANCE TO, INCLUDED
GABRB3	612269	EPILEPSY, CHILDHOOD ABSENCE, SUSCEPTIBILITY TO, 5; ECA5
GABRB3	617113	DEVELOPMENTAL AND EPILEPTIC ENCEPHALOPATHY 43; DEE43;;EPILEPTIC ENCEPHALOPATHY, EARLY INFANTILE, 43; EIEE43
GCNT2	110800	BLOOD GROUP, I SYSTEM; Ii;;I BLOOD GROUP SYSTEM;;Ii BLOOD GROUP SYSTEM ADULT i PHENOTYPE, INCLUDED
GCNT2	116700	CATARACT 13 WITH ADULT i PHENOTYPE; CTRCT13
GDAP1	214400	CHARCOT-MARIE-TOOTH DISEASE, TYPE 4A; CMT4A;;CHARCOT-MARIE-TOOTH DISEASE, DEMYELINATING, AUTOSOMAL RECESSIVE, TYPE 4A;;CHARCOT-MARIE-TOOTH NEUROPATHY, TYPE 4A
GDAP1	607706	CHARCOT-MARIE-TOOTH DISEASE, AXONAL, WITH VOCAL CORD PARESIS, AUTOSOMAL RECESSIVE;;CMT2 WITH VOCAL CORD PARESIS, AUTOSOMAL RECESSIVE;;CHARCOT-MARIE-TOOTH DISEASE, TYPE 4A, AXONAL FORM;;CHARCOT-MARIE-TOOTH NEUROPATHY, AXONAL, WITH VOCAL CORD PARESIS, /.../OMAL RECESSIVE
GDAP1	607831	CHARCOT-MARIE-TOOTH DISEASE, AXONAL, TYPE 2K; CMT2K;;CHARCOT-MARIE-TOOTH DISEASE, AXONAL, AUTOSOMAL RECESSIVE, TYPE 2K;;CHARCOT-MARIE-TOOTH NEUROPATHY, AXONAL, TYPE 2K CHARCOT-MARIE-TOOTH DISEASE, AUTOSOMAL DOMINANT, TYPE 2K, INCLUDED
GDAP1	608340	CHARCOT-MARIE-TOOTH DISEASE, RECESSIVE INTERMEDIATE A; CMTRIA;;CHARCOT-MARIE-TOOTH NEUROPATHY, RECESSIVE INTERMEDIATE A;;RI-CMTA

GDF11	619122	VERTEBRAL HYPERSEGMENTATION AND OROFACIAL ANOMALIES; VHO
GDI1	300849	INTELLECTUAL DEVELOPMENTAL DISORDER, X-LINKED 41; XLID41;;MENTAL RETARDATION, X-LINKED 41; MRX41;;MENTAL RETARDATION, X-LINKED 48; MRX48
GEMIN8	NA	
GIMAP1-GIMAP5	NA	
GIMAP5	619463	PORTAL HYPERTENSION, NONCIRRHOTIC, 2; NCPH2
GINS1	617827	IMMUNODEFICIENCY 55; IMD55
GIPC3	601869	DEAFNESS, AUTOSOMAL RECESSIVE 15; DFNB15;;DEAFNESS, AUTOSOMAL RECESSIVE 72; DFNB72;;DEAFNESS, AUTOSOMAL RECESSIVE 95; DFNB95
GLB1L3	NA	
GLP1R	NA	
GLRA2	301076	INTELLECTUAL DEVELOPMENTAL DISORDER, X-LINKED, SYNDROMIC, PILORGE TYPE; MRXSP
GLYATL1	NA	
GNB3	145500	HYPERTENSION, ESSENTIAL;;EHT
GNB3	617024	NIGHT BLINDNESS, CONGENITAL STATIONARY, TYPE 1H; CSNB1H
GPR37	NA	
GPR50	NA	
GPR68	617217	AMELOGENESIS IMPERFECTA, HYPOMATURATION TYPE, IIA6; AI2A6
GRIA3	300699	INTELLECTUAL DEVELOPMENTAL DISORDER, X-LINKED, SYNDROMIC, WU TYPE; MRXSW;;MENTAL RETARDATION, X-LINKED 94; MRX94;;MENTAL RETARDATION, X-LINKED, SYNDROMIC 29; MRXS29
GRIN1	614254	NEURODEVELOPMENTAL DISORDER WITH OR WITHOUT HYPERKINETIC MOVEMENTS AND SEIZURES, AUTOSOMAL DOMINANT; NDHMSD;;MENTAL RETARDATION, AUTOSOMAL DOMINANT 8, FORMERLY; MRD8, FORMERLY
GRIN1	617820	NEURODEVELOPMENTAL DISORDER WITH OR WITHOUT HYPERKINETIC MOVEMENTS AND SEIZURES, AUTOSOMAL RECESSIVE; NDHMSR
GRIN1	619814	DEVELOPMENTAL AND EPILEPTIC ENCEPHALOPATHY 101; DEE101
GRK2	NA	
GRK5	NA	
GTPBP2	617988	JABERI-ELAHI SYNDROME; JABELS
GTPBP6	NA	
H3C7	NA	
HADHA	609015	MITOCHONDRIAL TRIFUNCTIONAL PROTEIN DEFICIENCY 1; MTPD1;;MTPD;;TRIFUNCTIONAL PROTEIN DEFICIENCY MITOCHONDRIAL TRIFUNCTIONAL PROTEIN DEFICIENCY 1 WITH MYOPATHY AND NEUROPATHY, INCLUDED
HADHA	609016	LONG-CHAIN 3-HYDROXYACYL-CoA DEHYDROGENASE DEFICIENCY;;LCHAD DEFICIENCY
HERC2	176270	PRADER-WILLI SYNDROME; PWS;;PRADER-LABHART-WILLI SYNDROME PRADER-WILLI SYNDROME CHROMOSOME REGION, INCLUDED; PWCR, INCLUDED;;PRADER-WILLI-LIKE SYNDROME ASSOCIATED WITH CHROMOSOME 6, INCLUDED
HERC2	227220	SKIN/HAIR/EYE PIGMENTATION, VARIATION IN, 1; SHEP1;;SKIN/HAIR/EYE PIGMENTATION 1, BLUE/NONBLUE EYES;;SKIN/HAIR/EYE PIGMENTATION 1, BLUE/BROWN EYES;;SKIN/HAIR/EYE PIGMENTATION 1, BLOND/BROWN HAIR;;EYE COLOR, BROWN/BLUE;;EYE COLOR, BLUE/NONBLUE;;EYE C /.../; EYCL3;;BROWN EYE COLOR 2; BEY2;;HAIR COLOR 3; HCL3
HERC2	615516	INTELLECTUAL DEVELOPMENTAL DISORDER, AUTOSOMAL RECESSIVE 38; MRT38;;MENTAL RETARDATION, AUTOSOMAL RECESSIVE 38
HGD	203500	ALKAPTONURIA; AKU;;HOMOGENTISIC ACID OXIDASE DEFICIENCY
HIPK1	NA	
HMCN1	603075	MACULAR DEGENERATION, AGE-RELATED, 1; ARMD1;;MACULOPATHY, AGE-RELATED, 1
HNRNPF	NA	
HSPA5	NA	
HTATSF1	NA	
IFNA16	NA	
IGF2BP2	125853	TYPE 2 DIABETES MELLITUS; T2D;;DIABETES MELLITUS, NONINSULIN-DEPENDENT; NIDDM;;NONINSULIN-DEPENDENT DIABETES MELLITUS;;DIABETES MELLITUS, TYPE II;;MATURITY-ONSET DIABETES INSULIN RESISTANCE, SUSCEPTIBILITY TO, INCLUDED;;DIABETES MELLITUS, TYPE 2, PR /.../ON AGAINST, INCLUDED
IGF2R	114550	HEPATOCELLULAR CARCINOMA;;HCC;;CANCER, HEPATOCELLULAR;;LIVER CANCER;;LIVER CELL CARCINOMA; LCC;;HEPATOMA HEPATOBLASTOMA, INCLUDED;;HEPATOBLASTOMA CAUSED BY SOMATIC MUTATION, INCLUDED
IGFBP4	NA	
IGFN1	NA	
IGSF1	300888	HYPOTHYROIDISM, CENTRAL, WITH TESTICULAR ENLARGEMENT; CHTE
IGSF9	NA	
IK	NA	
IKBKG	300291	ECTODERMAL DYSPLASIA AND IMMUNODEFICIENCY 1; EDAID1;;ECTODERMAL DYSPLASIA, ANHIDROTIC, WITH IMMUNODEFICIENCY, OSTEOPETROSIS, AND LYMPHEDEMA; OLEDAID;;ECTODERMAL DYSPLASIA, ANHIDROTIC, WITH IMMUNE DEFICIENCY;;ECTODERMAL DYSPLASIA, HYPOHIDROTIC, WITH /.../ DEFICIENCY; HEDID;;HYPER-IgM IMMUNODEFICIENCY, X-LINKED, WITH HYPOHIDROTIC ECTODERMAL DYSPLASIA; XHMED
IKBKG	300636	IMMUNODEFICIENCY 33; IMD33;;INVASIVE PNEUMOCOCCAL DISEASE, RECURRENT ISOLATED, 2, FORMERLY; IPD2, FORMERLY
IKBKG	301081	AUTOINFLAMMATORY DISEASE, SYSTEMIC, X-LINKED; SAIDX
IKBKG	308300	INCONTINENTIA PIGMENTI; IP;;INCONTINENTIA PIGMENTI, FAMILIAL MALE-LETHAL TYPE;;BLOCH-SULZBERGER SYNDROME;;INCONTINENTIA PIGMENTI, TYPE II, FORMERLY; IP2, FORMERLY
IL1RAPL2	NA	
IL20RA	NA	
INTS5	NA	
IQCE	617642	POLYDACTYLY, POSTAXIAL, TYPE A7; PAPA7

IRAK2	NA	
IRS4	301035	HYPOTHYROIDISM, CONGENITAL, NONGOITROUS, 9; CHNG9
ITGA5	NA	
ITPRIPL2	NA	
JMJD4	NA	
JUP	601214	NAXOS DISEASE; NXD;;CARDIOMYOPATHY, ARRHYTHMOGENIC RIGHT VENTRICULAR, WITH SKIN, HAIR, AND NAIL ABNORMALITIES;;MAL DE NAXOS;;KERATOSIS PALMOPLANTARIS WITH ARRHYTHMOGENIC CARDIOMYOPATHY;;WOOLLY HAIR, PALMOPLANTAR KERATODERMA, AND CARDIAC ABNORMALITIE /.../ MOPLANTAR KERATODERMA WITH ARRHYTHMOGENIC RIGHT VENTRICULAR CARDIOMYOPATHY AND WOOLLY HAIR
JUP	611528	ARRHYTHMOGENIC RIGHT VENTRICULAR DYSPLASIA, FAMILIAL, 12; ARVD12;;ARRHYTHMOGENIC RIGHT VENTRICULAR CARDIOMYOPATHY 12; ARVC12
KCNA5	612240	ATRIAL FIBRILLATION, FAMILIAL, 7; ATFB7
KCNA7	NA	
KCNAB2	NA	
KCNAB3	NA	
KCNG2	NA	
KCNN4	616689	DEHYDRATED HEREDITARY STOMATOCYTOSIS 2; DHS2;;XEROCYTOSIS GARDOS;;DESICCYTOSIS GARDOS
KDM5A	NA	
KDM5B	618109	INTELLECTUAL DEVELOPMENTAL DISORDER, AUTOSOMAL RECESSIVE 65; MRT65;;MENTAL RETARDATION, AUTOSOMAL RECESSIVE 65
KHDC1L	NA	
KIAA1549L	NA	
KIF18A	NA	
KIF26B	NA	
KIF4A	300923	INTELLECTUAL DEVELOPMENTAL DISORDER, X-LINKED 100; XLID100;;MENTAL RETARDATION, X-LINKED 100; MRX100
KIF4A	313490	TAURODONTISM, MICRODONTIA, AND DENS INVAGINATUS; TMDI
KIF7	200990	ACROCALLOSAL SYNDROME; ACLS;;HALLUX DUPLICATION, POSTAXIAL POLYDACTYLY, AND ABSENCE OF CORPUS CALLOSUM;;SCHINZEL ACROCALLOSAL SYNDROME JOUBERT SYNDROME 12, INCLUDED; JBTS12, INCLUDED;;JOUBERT SYNDROME 12/15, DIGENIC, INCLUDED
KIF7	607131	AL-GAZALI-BAKALINOVA SYNDROME; AGBK;;MACROCEPHALY WITH MULTIPLE EPIPHYSEAL DYSPLASIA AND DISTINCTIVE FACIES; MMEDF
KIF7	614120	HYDROLETHALUS SYNDROME 2; HLS2
KLF17	NA	
KLHL7	612943	RETINITIS PIGMENTOSA 42; RP42
KLHL7	617055	PERCHING SYNDROME; PERCHING;;CRISPONI/COLD-INDUCED SWEATING SYNDROME 3, FORMERLY; CISS3, FORMERLY
KLRG2	NA	
KMT2B	617284	DYSTONIA 28, CHILDHOOD-ONSET; DYT28
KMT2B	619934	INTELLECTUAL DEVELOPMENTAL DISORDER, AUTOSOMAL DOMINANT 68; MRD68;;MENTAL RETARDATION, AUTOSOMAL DOMINANT 68
KRT38	NA	
KTN1	NA	
L1CAM	303350	MASA SYNDROME;;MENTAL RETARDATION, APHASIA, SHUFFLING GAIT, AND ADDUCTED THUMBS;;SPASTIC PARAPLEGIA 1, X-LINKED; SPG1;;CLASPED THUMB AND MENTAL RETARDATION;;THUMB, CONGENITAL CLASPED, WITH MENTAL RETARDATION;;ADDUCTED THUMB WITH MENTAL RETARDATION;; /.../-MASON SYNDROME;;CRASH SYNDROME
L1CAM	304100	CORPUS CALLOSUM, PARTIAL AGENESIS OF, X-LINKED
L1CAM	307000	HYDROCEPHALUS, CONGENITAL, X-LINKED; HYCX;;HYDROCEPHALUS DUE TO CONGENITAL STENOSIS OF AQUEDUCT OF SYLVIUS; HSAS;;HSAS1;;HYDROCEPHALUS, X-LINKED;;AQUEDUCTAL STENOSIS, X-LINKED; XLAS
L3MBTL1	NA	
LGALS9B	NA	
LILRA1	NA	
LIMCH1	NA	
LMTK3	NA	
LPL	144250	HYPERLIPIDEMIA, FAMILIAL COMBINED, 3; FCHL3;;FAMILIAL COMBINED HYPERLIPIDEMIA
LPL	238600	HYPERLIPOPROTEINEMIA, TYPE I;;LIPOPROTEIN LIPASE DEFICIENCY;;LPL DEFICIENCY;;HYPERCHYLOMICRONEMIA, FAMILIAL;;HYPERLIPEMIA, IDIOPATHIC, BURGER-GRUTZ TYPE;;HYPERLIPEMIA, ESSENTIAL FAMILIAL;;LIPASE D DEFICIENCY;;LIPD DEFICIENCY;;HYPERLIPOPROTEINEMIA, T /.../;;CHYLOMICRONEMIA, FAMILIAL HIGH DENSITY LIPOPROTEIN CHOLESTEROL LEVEL QUANTITATIVE TRAIT LOCUS 11, INCLUDED; HDLCQ11, INCLUDED
LRBA	614700	IMMUNODEFICIENCY, COMMON VARIABLE, 8, WITH AUTOIMMUNITY; CVID8
LRIG1	NA	
LRP1	604093	KERATOSIS PILARIS ATROPHICANS; KPA
LRRC34	NA	
LRRN2	NA	
MAGEC1	NA	
MANSC1	NA	
MAP3K3	NA	
MARCHF7	NA	
MCF2L	NA	
MDN1	NA	
MEGF8	614976	CARPENTER SYNDROME 2; CRPT2
MEI1	618431	HYDATIDIFORM MOLE, RECURRENT, 3; HYDM3

MEN1	131100	MULTIPLE ENDOCRINE NEOPLASIA, TYPE I; MEN1;;MEN I;;ENDOCRINE ADENOMATOSIS, MULTIPLE;;MEA I;;WERMER SYNDROME MEN1 SOMATIC MUTATIONS, INCLUDED
METTL24	NA	
METTL25	NA	
MGAT5B	NA	
MMP2	259600	MULTICENTRIC OSTEOLYSIS, NODULOSIS, AND ARTHROPATHY; MONA;;TORG SYNDROME;;NODULOSIS-ARTHROPATHY-OSTEOLYSIS SYNDROME;;NAO SYNDROME;;AL-AQEEL SEWAIRI SYNDROME;;OSTEOLYSIS, HEREDITARY MULTICENTRIC;;TORG-WINCHESTER SYNDROME, FORMERLY
MMP26	NA	
MPI	602579	CONGENITAL DISORDER OF GLYCOSYLATION, TYPE Ib; CDG1B;;CDG Ib; CDGIb;;CDG, GASTROINTESTINAL TYPE;;MANNOSEPHOSPHATE ISOMERASE DEFICIENCY;;MPI DEFICIENCY;;PROTEIN-LOSING ENTEROPATHY-HEPATIC FIBROSIS SYNDROME;;SAGUENAY-LAC SAINT-JEAN SYNDROME;;SLSJ SYNDROME
MROH2B	NA	
MSC	NA	
MT1H	NA	
MTTP	200100	ABETALIPOPROTEINEMIA; ABL;;ACANTHOCYTOSIS;;BASSEN-KORNZWEIG SYNDROME;;MICROSOMAL TRIGLYCERIDE TRANSFER PROTEIN DEFICIENCY;;MTP DEFICIENCY
MUC16	NA	
MUC5AC	NA	
MUSK	208150	FETAL AKINESIA DEFORMATION SEQUENCE 1; FADS1;;FETAL AKINESIA DEFORMATION SEQUENCE; FADS;;PENA-SHOKEIR SYNDROME, TYPE I;;FETAL AKINESIA SEQUENCE;;ARTHROGRYPOSIS MULTIPLEX CONGENITA WITH PULMONARY HYPOPLASIA
MUSK	616325	MYASTHENIC SYNDROME, CONGENITAL, 9, ASSOCIATED WITH ACETYLCHOLINE RECEPTOR DEFICIENCY; CMS9
MYBL2	NA	
MYH1	NA	
MYH13	NA	
MYH4	NA	
MYH7	160500	MYOPATHY, DISTAL, 1; MPD1;;MYOPATHY, LATE DISTAL HEREDITARY;;LAING DISTAL MYOPATHY;;MYOPATHY, DISTAL, EARLY-ONSET, AUTOSOMAL DOMINANT
MYH7	192600	CARDIOMYOPATHY, FAMILIAL HYPERTROPHIC, 1; CMH1;;CMH;;VENTRICULAR HYPERTROPHY, HEREDITARY;;ASYMMETRIC SEPTAL HYPERTROPHY; ASH;;HYPERTROPHIC SUBAORTIC STENOSIS, IDIOPATHIC
MYH7	255160	CONGENITAL MYOPATHY 7B, MYOSIN STORAGE, AUTOSOMAL RECESSIVE; CMYP7B;;MYOPATHY, MYOSIN STORAGE, AUTOSOMAL RECESSIVE; MSMB;;MYOPATHY, HYALINE BODY, AUTOSOMAL RECESSIVE
MYH7	608358	CONGENITAL MYOPATHY 7A, MYOSIN STORAGE, AUTOSOMAL DOMINANT; CMYP7A;;MYOPATHY, MYOSIN STORAGE, AUTOSOMAL DOMINANT; MSMA;;MYOPATHY, HYALINE BODY, AUTOSOMAL DOMINANT;;MYOPATHY WITH LYSIS OF TYPE I MYOFIBRILS;;SCAPULOPERONEAL MYOPATHY, MYH7-RELATED; SPM /.../PULOPERONEAL MUSCULAR DYSTROPHY; SPMD;;SCAPULOPERONEAL SYNDROME, MYOPATHIC TYPE
MYH7	613426	CARDIOMYOPATHY, DILATED, 1S; CMD1S LEFT VENTRICULAR NONCOMPACTION 5, INCLUDED; LVNC5, INCLUDED
MYO18B	616549	KLIPPEL-FEIL SYNDROME 4, AUTOSOMAL RECESSIVE, WITH NEMALINE MYOPATHY AND FACIAL DYSMORPHISM; KFS4
NALCN	615419	HYPOTONIA, INFANTILE, WITH PSYCHOMOTOR RETARDATION AND CHARACTERISTIC FACIES 1; IHPRF1;;IHPRF
NALCN	616266	CONGENITAL CONTRACTURES OF THE LIMBS AND FACE, HYPOTONIA, AND DEVELOPMENTAL DELAY; CLIFAHDD
NBEAL2	139090	GRAY PLATELET SYNDROME; GPS;;BLEEDING DISORDER, PLATELET-TYPE, 4; BDPLT4;;PLATELET ALPHA-GRANULE DEFICIENCY
NCEH1	NA	
NDUFA11	618236	MITOCHONDRIAL COMPLEX I DEFICIENCY, NUCLEAR TYPE 14; MC1DN14
NEBL	NA	
NECTIN1	225060	CLEFT LIP/PALATE-ECTODERMAL DYSPLASIA SYNDROME; CLPED1;;ZLOTOGORA-OGUR SYNDROME;;ECTODERMAL DYSPLASIA, MARGARITA ISLAND TYPE;;ECTODERMAL DYSPLASIA, TYPE 4; ED4;;ECTODERMAL DYSPLASIA, CLEFT LIP AND PALATE, MENTAL RETARDATION, AND SYNDACTYLY OROFACIAL /.../ 7, INCLUDED; OFC7, INCLUDED;;CLEFT LIP WITH OR WITHOUT CLEFT PALATE, NONSYNDROMIC, 7, INCLUDED
NEDD4L	617201	PERIVENTRICULAR NODULAR HETEROTOPIA 7; PVNH7
NEFM	NA	
NEUROD4	NA	
NFATC3	NA	
NFKBIA	612132	ECTODERMAL DYSPLASIA AND IMMUNODEFICIENCY 2; EDAID2;;ECTODERMAL DYSPLASIA, ANHIDROTIC, WITH IMMUNODEFICIENCY 2;;ECTODERMAL DYSPLASIA, HYPOHIDROTIC, WITH IMMUNODEFICIENCY 2;;ECTODERMAL DYSPLASIA, ANHIDROTIC, WITH T-CELL IMMUNODEFICIENCY, AUTOSOMAL DOMINANT
NHLRC3	NA	
NLRP2	620332	OOCYTE/ZYGOTE/EMBRYO MATURATION ARREST 18; OZEMA18
NLRP5	620333	OOCYTE/ZYGOTE/EMBRYO MATURATION ARREST 19; OZEMA19
NMU	NA	
NPAP1	176270	PRADER-WILLI SYNDROME; PWS;;PRADER-LABHART-WILLI SYNDROME PRADER-WILLI SYNDROME CHROMOSOME REGION, INCLUDED; PWCR, INCLUDED;;PRADER-WILLI-LIKE SYNDROME ASSOCIATED WITH CHROMOSOME 6, INCLUDED
NR4A2	168600	PARKINSON DISEASE, LATE-ONSET; PD;;PARK
NR4A2	619911	INTELLECTUAL DEVELOPMENTAL DISORDER WITH LANGUAGE IMPAIRMENT AND EARLY-ONSET DOPA-RESPONSIVE DYSTONIA-PARKINSONISM; IDLDP
NRIP1	NA	
NRXN1	614325	PITT-HOPKINS-LIKE SYNDROME 2; PTHSL2

NRXN1	614332	CHROMOSOME 2p16.3 DELETION SYNDROME SCHIZOPHRENIA 17, INCLUDED; SCZD17, INCLUDED
NUAK1	NA	
NUDT16L1	NA	
NUP205	616893	NEPHROTIC SYNDROME, TYPE 13; NPHS13
NUP37	618179	MICROCEPHALY 24, PRIMARY, AUTOSOMAL RECESSIVE; MCPH24
OAS2	NA	
OBSL1	612921	THREE M SYNDROME 2; 3M2;;3@M SYNDROME 2
OPN4	NA	
OR10H1	NA	
OR1L8	NA	
OR2T6	NA	
OR51E2	NA	
OR51L1	NA	
OR52B4	NA	
OTOG	614945	DEAFNESS, AUTOSOMAL RECESSIVE 18B; DFNB18B
OXSR1	NA	
PARP10	NA	
PCDH20	NA	
PCDHA1	NA	
PCDHA10	NA	
PCDHA11	NA	
PCDHA12	NA	
PCDHA13	NA	
PCDHA2	NA	
PCDHA3	NA	
PCDHA4	NA	
PCDHA5	NA	
PCDHA6	NA	
PCDHA7	NA	
PCDHA8	NA	
PCDHA9	NA	
PCDHAC1	NA	
PCDHAC2	NA	
PCDHGA1	NA	
PCDHGA10	NA	
PCDHGA2	NA	
PCDHGA3	NA	
PCDHGA4	NA	
PCDHGA5	NA	
PCDHGA6	NA	
PCDHGA7	NA	
PCDHGA8	NA	
PCDHGA9	NA	
PCDHGB1	NA	
PCDHGB2	NA	
PCDHGB3	NA	
PCDHGB4	NA	
PCDHGB5	NA	
PCDHGB6	NA	
PCK1	261680	PHOSPHOENOLPYRUVATE CARBOXYKINASE DEFICIENCY, CYTOSOLIC; PCKDC;;PCK1 DEFICIENCY, CYTOSOLIC;;PEPCK DEFICIENCY, CYTOSOLIC
PDE7B	NA	
PGS1	NA	
PIEZO2	108145	ARTHROGRYPOSIS, DISTAL, TYPE 5; DA5;;ARTHROGRYPOSIS WITH OCULOMOTOR LIMITATION AND ELECTRORETINAL ABNORMALITIES;;OCULOMELIC AMYOPLASIA;;ARTHROGRYPOSIS, DISTAL, TYPE IIB; DAIIB
PIEZO2	114300	ARTHROGRYPOSIS, DISTAL, TYPE 3; DA3;;GORDON SYNDROME;;ARTHROGRYPOSIS MULTIPLEX CONGENITA, DISTAL, TYPE IIA;;CAMPTODACTYLY, CLEFT PALATE, AND CLUBFOOT
PIEZO2	248700	MARDEN-WALKER SYNDROME; MWKS;;MWS
PIEZO2	617146	ARTHROGRYPOSIS, DISTAL, WITH IMPAIRED PROPRIOCEPTION AND TOUCH; DAIPT
PIKFYVE	121850	CORNEAL DYSTROPHY, FLECK; CFD;;FLECK CORNEAL DYSTROPHY; FCD;;CORNEAL DYSTROPHY, FRANCOIS-NEETENS SPECKLED OR FLECKED
PKHD1	263200	POLYCYSTIC KIDNEY DISEASE 4 WITH OR WITHOUT POLYCYSTIC LIVER DISEASE; PKD4;;POLYCYSTIC KIDNEY DISEASE 4 WITH OR WITHOUT HEPATIC DISEASE;;POLYCYSTIC KIDNEY DISEASE, AUTOSOMAL RECESSIVE; ARPKD;;POLYCYSTIC KIDNEY AND HEPATIC DISEASE 1; PKHD1;;POLYCYSTI /.../EY DISEASE, INFANTILE, TYPE I;;PKD3, FORMERLY HEPATIC FIBROSIS, CONGENITAL, INCLUDED
PKN2	NA	
PLA2G15	NA	
PLAAT1	NA	
POU4F3	602459	DEAFNESS, AUTOSOMAL DOMINANT 15; DFNA15
PPIF	NA	
PPP3CC	NA	
PRAME	NA	
PRAMEF17	NA	

PRKAG2	194200	WOLFF-PARKINSON-WHITE SYNDROME; WPW;;WPW SYNDROME PREEXCITATION SYNDROME, INCLUDED;;ACCESSORY ATRIOVENTRICULAR PATHWAYS, INCLUDED
PRKAG2	261740	GLYCOGEN STORAGE DISEASE OF HEART, LETHAL CONGENITAL;;PHOSPHORYLASE KINASE DEFICIENCY OF HEART;;GLYCOGEN STORAGE DISEASE OF HEART
PRKAG2	600858	CARDIOMYOPATHY, FAMILIAL HYPERTROPHIC, 6; CMH6
PRSS12	249500	INTELLECTUAL DEVELOPMENTAL DISORDER, AUTOSOMAL RECESSIVE 1; MRT1;;MENTAL RETARDATION, AUTOSOMAL RECESSIVE 1
PRSS54	NA	
PRSS8	NA	
PSMD13	NA	
PSMD2	NA	
PSMD9	NA	
PTK7	NA	
PTPRF	616001	BREASTS AND/OR NIPPLES, APLASIA OR HYPOPLASIA OF, 2; BNAH2
PTPRU	NA	
PWP2	NA	
PWWP3B	NA	
PXDNL	NA	
RAB6D	NA	
RAD51AP1	NA	
RALGDS	NA	
RAPGEF6	NA	
RASD2	NA	
RASGRP4	NA	
RASIP1	NA	
RBAK	NA	
RBAK-RBAKDN	NA	
RBKS	NA	
RBPJ	614814	ADAMS-OLIVER SYNDROME 3; AOS3
REG1A	NA	
RIMS1	NA	
RMDN2	NA	
ROBO1	257400	NYSTAGMUS 8, CONGENITAL, AUTOSOMAL RECESSIVE; NYS8
ROBO1	620303	PITUITARY HORMONE DEFICIENCY, COMBINED OR ISOLATED, 8; CPHD8
ROBO1	620305	NEUROOCULORENAL SYNDROME; NORS
ROBO4	618496	AORTIC VALVE DISEASE 3; AOVD3;;BICUSPID AORTIC VALVE;;AORTIC VALVE STENOSIS
ROS1	NA	
RP1	180100	RETINITIS PIGMENTOSA 1; RP1
RSL1D1	NA	
RYR3	620310	CONGENITAL MYOPATHY 20; CMYP20
S100A11	NA	
SALL3	NA	
SCN10A	615551	EPISODIC PAIN SYNDROME, FAMILIAL, 2; FEPS2
SELP	NA	
SEMA6C	NA	
SERPINB11	NA	
SERPINE1	613329	PLASMINOGEN ACTIVATOR INHIBITOR-1 DEFICIENCY;;HYPERFIBRINOLYSIS DUE TO PAI1 DEFICIENCY
SETD1B	619000	INTELLECTUAL DEVELOPMENTAL DISORDER WITH SEIZURES AND LANGUAGE DELAY; IDDSELD
SGTA	NA	
SH2D3C	NA	
SH2D7	NA	
SHISA2	NA	
SIDT1	NA	
SLC12A5	616645	DEVELOPMENTAL AND EPILEPTIC ENCEPHALOPATHY 34; DEE34;;EPILEPTIC ENCEPHALOPATHY, EARLY INFANTILE, 34; EIEE34
SLC12A5	616685	EPILEPSY, IDIOPATHIC GENERALIZED, SUSCEPTIBILITY TO, 14; EIG14
SLC28A1	618477	URIDINE-CYTIDINEURIA; URCTU
SLC32A1	NA	
SLC34A1	612286	NEPHROLITHIASIS/OSTEOPOROSIS, HYPOPHOSPHATEMIC, 1; NPHLOP1
SLC34A1	613388	FANCONI RENOTUBULAR SYNDROME 2; FRTS2
SLC34A1	616963	HYPERCALCEMIA, INFANTILE, 2; HCINF2
SLC34A2	265100	PULMONARY ALVEOLAR MICROLITHIASIS; PULAM
SLC7A6	NA	
SLC9A5	NA	
SLIT1	NA	
SMAP1	NA	
SMARCA2	601358	NICOLAIDES-BARAITSER SYNDROME; NCBRS;;SPARSE HAIR-IMPAIRED INTELLECTUAL DEVELOPMENT SYNDROME;;NBS
SMARCA2	619293	BLEPHAROPHIMOSIS-IMPAIRED INTELLECTUAL DEVELOPMENT SYNDROME; BIS
SMARCC2	618362	COFFIN-SIRIS SYNDROME 8; CSS8
SMC4	NA	
SMC5	620185	ATELIS SYNDROME 2; ATELS2;;POOR GROWTH, MICROCEPHALY, DYSMORPHIC FACIES, AND CARDIAC DEFECTS;;MOSAIC VARIEGATED ANEUPLOIDY SYNDROME 6; MVA6
SMYD5	NA	

SNAP47	NA	
SORT1	613589	LOW DENSITY LIPOPROTEIN CHOLESTEROL LEVEL QUANTITATIVE TRAIT LOCUS 6; LDLCQ6
SP8	NA	
SPAG5	NA	
SPATA31E1	NA	
SPIRE2	NA	
SSH1	NA	
ST3GAL2	NA	
STAC	NA	
STARD8	NA	
STK17A	NA	
STPG2	NA	
SUPT16H	619480	NEURODEVELOPMENTAL DISORDER WITH DYSMORPHIC FACIES AND THIN CORPUS CALLOSUM; NEDDFAC
SVEP1	NA	
TAF7L	NA	
TAFA3	NA	
TANGO6	NA	
TBC1D13	NA	
TBC1D16	NA	
TBPL2	NA	
TBX4	147891	ISCHIOCOXOPODOPATELLAR SYNDROME WITH OR WITHOUT PULMONARY ARTERIAL HYPERTENSION; ICPPS;;SMALL PATELLA SYNDROME; SPS;;PATELLA APLASIA, COXA VARA, AND TARSAL SYNOSTOSIS;;ISCHIOPATELLAR DYSPLASIA;;COXOPODOPATELLAR SYNDROME;;SCOTT-TAOR SYNDROME
TBX4	601360	AMELIA, POSTERIOR, WITH PELVIC AND PULMONARY HYPOPLASIA SYNDROME; PAPPAS;;AMELIA, AUTOSOMAL RECESSIVE
TCF7L2	125853	TYPE 2 DIABETES MELLITUS; T2D;;DIABETES MELLITUS, NONINSULIN-DEPENDENT; NIDDM;;NONINSULIN-DEPENDENT DIABETES MELLITUS;;DIABETES MELLITUS, TYPE II;;MATURITY-ONSET DIABETES INSULIN RESISTANCE, SUSCEPTIBILITY TO, INCLUDED;;DIABETES MELLITUS, TYPE 2, PR /.../ON AGAINST, INCLUDED
TDRD10	NA	
TENM4	616736	TREMOR, HEREDITARY ESSENTIAL, 5; ETM5
TEP1	NA	
TFDP3	NA	
TIMM50	617698	3-@METHYLGLUTACONIC ACIDURIA, TYPE IX; MGCA9
TMEM132D	NA	
TMEM151A	620245	EPISODIC KINESIGENIC DYSKINESIA 3; EKD3;;DYSTONIA 36; DYT36
TMEM163	620243	LEUKODYSTROPHY, HYPOMYELINATING, 25; HLD25
TMEM175	NA	
TMEM219	NA	
TMEM220	NA	
TMEM95	NA	
TNFAIP3	NA	
TNPO2	619556	INTELLECTUAL DEVELOPMENTAL DISORDER WITH HYPOTONIA, IMPAIRED SPEECH, AND DYSMORPHIC FACIES; IDDHISD
TP53AIP1	NA	
TP53BP1	NA	
TRIM65	NA	
TSPOAP1	620453	DYSTONIA 22, JUVENILE-ONSET; DYT22JO
TSPOAP1	620456	DYSTONIA 22, ADULT-ONSET; DYT22AO
TSSC4	NA	
TTC21B	613819	SHORT-RIB THORACIC DYSPLASIA 4 WITH OR WITHOUT POLYDACTYLY; SRTD4;;ASPHYXIATING THORACIC DYSTROPHY 4; ATD4
TTC21B	613820	NEPHRONOPHTHISIS 12; NPHP12 JOUBERT SYNDROME 11, INCLUDED; JBTS11, INCLUDED
TTC39A	NA	
TTN	600334	TIBIAL MUSCULAR DYSTROPHY, TARDIVE; TMD;;TARDIVE TIBIAL MUSCULAR DYSTROPHY;;UDD MYOPATHY
TTN	603689	MYOPATHY, MYOFIBRILLAR, 9, WITH EARLY RESPIRATORY FAILURE; MFM9;;HEREDITARY MYOPATHY WITH EARLY RESPIRATORY FAILURE; HMERF;;MYOPATHY, PROXIMAL, WITH EARLY RESPIRATORY MUSCLE INVOLVEMENT; MPRM;;EDSTROM MYOPATHY;;MYOPATHY, DISTAL, WITH EARLY RESPIRATO /.../ LURE, AUTOSOMAL DOMINANT
TTN	604145	CARDIOMYOPATHY, DILATED, 1G; CMD1G
TTN	608807	MUSCULAR DYSTROPHY, LIMB-GIRDLE, AUTOSOMAL RECESSIVE 10; LGMDR10;;MUSCULAR DYSTROPHY, LIMB-GIRDLE, TYPE 2J; LGMD2J
TTN	611705	CONGENITAL MYOPATHY 5 WITH CARDIOMYOPATHY; CMYP5;;SALIH MYOPATHY; SALMY;;MYOPATHY, EARLY-ONSET, WITH FATAL CARDIOMYOPATHY; EOMFC
TTN	613765	CARDIOMYOPATHY, FAMILIAL HYPERTROPHIC, 9; CMH9
TUBB2A	615763	CORTICAL DYSPLASIA, COMPLEX, WITH OTHER BRAIN MALFORMATIONS 5; CDCBM5
TULP4	NA	
U2AF1	NA	
UBA6	NA	
UBR1	243800	JOHANSON-BLIZZARD SYNDROME; JBS;;NASAL ALAR HYPOPLASIA, HYPOTHYROIDISM, PANCREATIC ACHYLIA, AND CONGENITAL DEAFNESS
UNC50	NA	
UNC5A	NA	

UNC5B	NA	
UNC80	616801	HYPOTONIA, INFANTILE, WITH PSYCHOMOTOR RETARDATION AND CHARACTERISTIC FACIES 2; IHPRF2
USP32	NA	
USP42	NA	
UTP20	NA	
VARS2	615917	COMBINED OXIDATIVE PHOSPHORYLATION DEFICIENCY 20; COXPD20
VGLL1	NA	
VIRMA	NA	
VPS41	619389	SPINOCEREBELLAR ATAXIA, AUTOSOMAL RECESSIVE 29; SCAR29;;BARAKAT-VAN HAM-KAYA SYNDROME; BAVAHAKA;;NEURODEVELOPMENTAL DISORDER WITH HYPOTONIA AND CEREBELLAR ATAXIA; NEDHCA
VSIG4	NA	
VWA3B	616948	SPINOCEREBELLAR ATAXIA, AUTOSOMAL RECESSIVE 22; SCAR22
VWA5B2	NA	
VWDE	NA	
WDCP	NA	
WDR13	NA	
YES1	NA	
ZBTB25	NA	
ZFX	NA	
ZHX2	NA	
ZMYND12	NA	
ZNF280C	NA	
ZNF326	NA	
ZNF440	NA	
ZNF446	NA	
ZNF536	NA	
ZNF564	NA	
ZNF584	NA	
ZNF587B	NA	
ZNF608	NA	
ZNF648	NA	
ZNF665	NA	
ZNF721	NA	
ZNF738	NA	
ZNF776	NA	
ZNF800	NA	
ZNF804A	NA	
ZNF829	NA	
ZNRF4	NA	
ZSCAN32	NA	

Supplemental Table S5

patient	tissue	pos	gene	ref_alt	ref_reads	mut_reads	vaf	cell frequency	effect_combined
**8**	buffy coat	4:105269613	*TET2*	G/T	458	96	0.173	0.347	LoF
**5**	buffy coat	1:120069404	*NOTCH2*	C/T	929	113	0.108	0.217	LoF
**13**	buffy coat	20:32434630	*ASXL1*	GCCATCGGAGG/-	1214	126	0.094	0.188	LoF
**13**	buffy coat	1:114716123	*NRAS*	C/T	907	88	0.088	0.177	Missense
**13**	buffy coat	17:7674918	*TP53*	A/T	1072	64	0.056	0.113	Missense
**7**	buffy coat	17:7675075	*TP53*	A/C	549	30	0.052	0.104	Missense
**12**	buffy coat	4:105272723	*TET2*	GTTCTTTTCGGCGAA	866	46	0.050	0.101	LoF
**8**	buffy coat	4:105235860	*TET2*	C/T	852	45	0.050	0.100	LoF
**7**	buffy coat	12:22659060	*ETNK1*	A/G	550	28	0.048	0.097	Missense
**7**	buffy coat	16:67620740	*CTCF*	G/A	415	11	0.026	0.052	Missense
**13**	buffy coat	2:25240439	*DNMT3A*	G/A	1210	32	0.026	0.052	Missense
**7**	buffy coat	3:47123693	*SETD2*	C/A	436	10	0.022	0.045	LoF
**13**	buffy coat	17:60663083	*PPM1D*	T/G	1122	25	0.022	0.044	LoF
**11**	buffy coat	2:25234373	*DNMT3A*	C/T	512	11	0.021	0.042	Missense
**13**	buffy coat	4:105276158	*TET2*	C/G	1346	27	0.020	0.039	Missense
**11**	buffy coat	2:25234307	*DNMT3A*	G/A	503	8	0.016	0.031	Missense
**13**	buffy coat	7:102196778	*CUX1*	T/G	1358	18	0.013	0.026	LoF

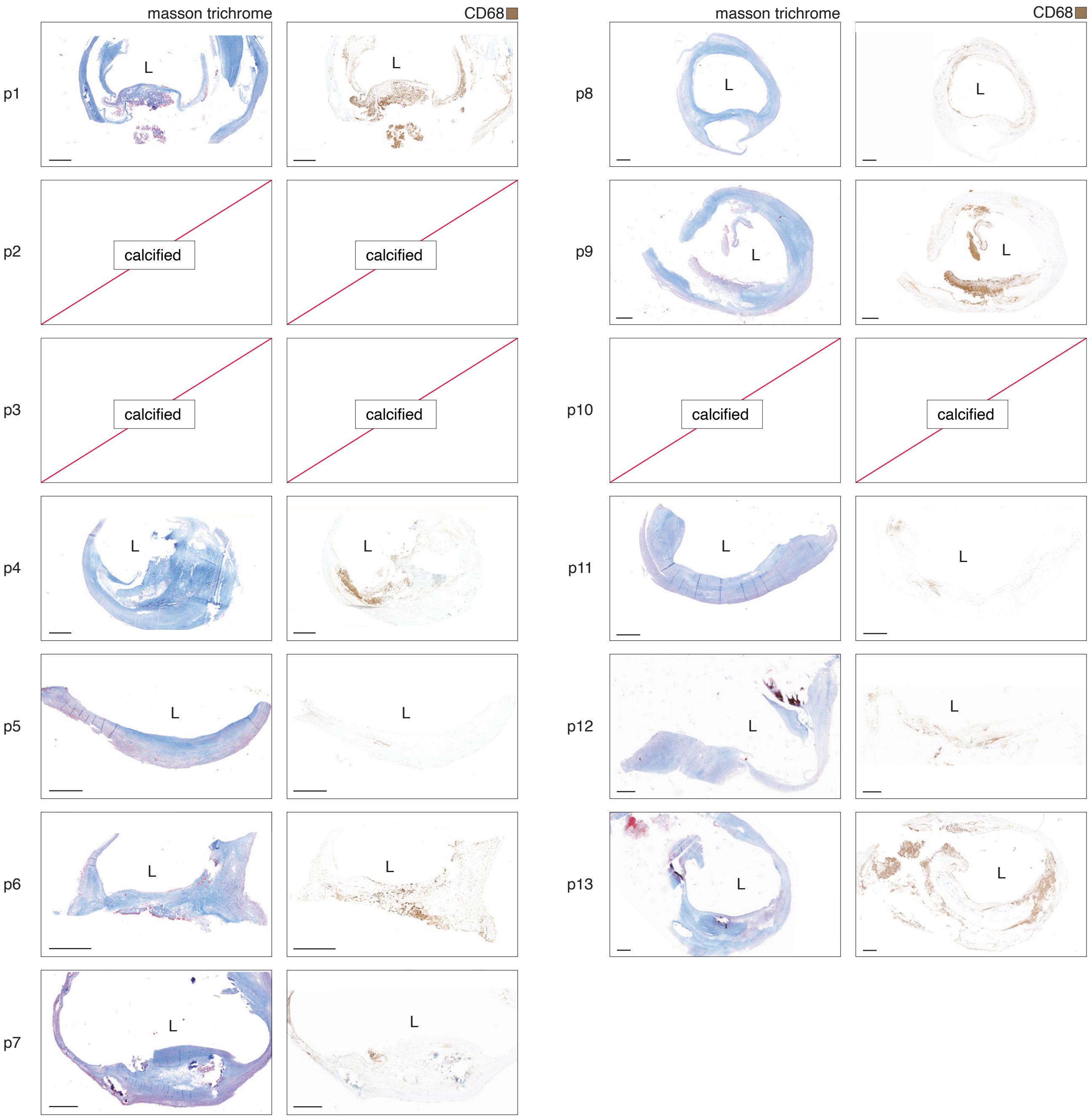

Supplemental Figure S1 I Histological representation of 10 of the 13 plaques

Formalin-fixed paraffin-embedded plaque segments from 10 of the 13 carotid plaques included in the study were sectioned and stained using Masson's trichrome, and immunohistochemically stained for the macrophage marker, CD68. FFPE blocks from patients 2, 3, and 10 were too calcified to generate histological sections. L indicates the lumen. Scalebars correspond to 1 mm.

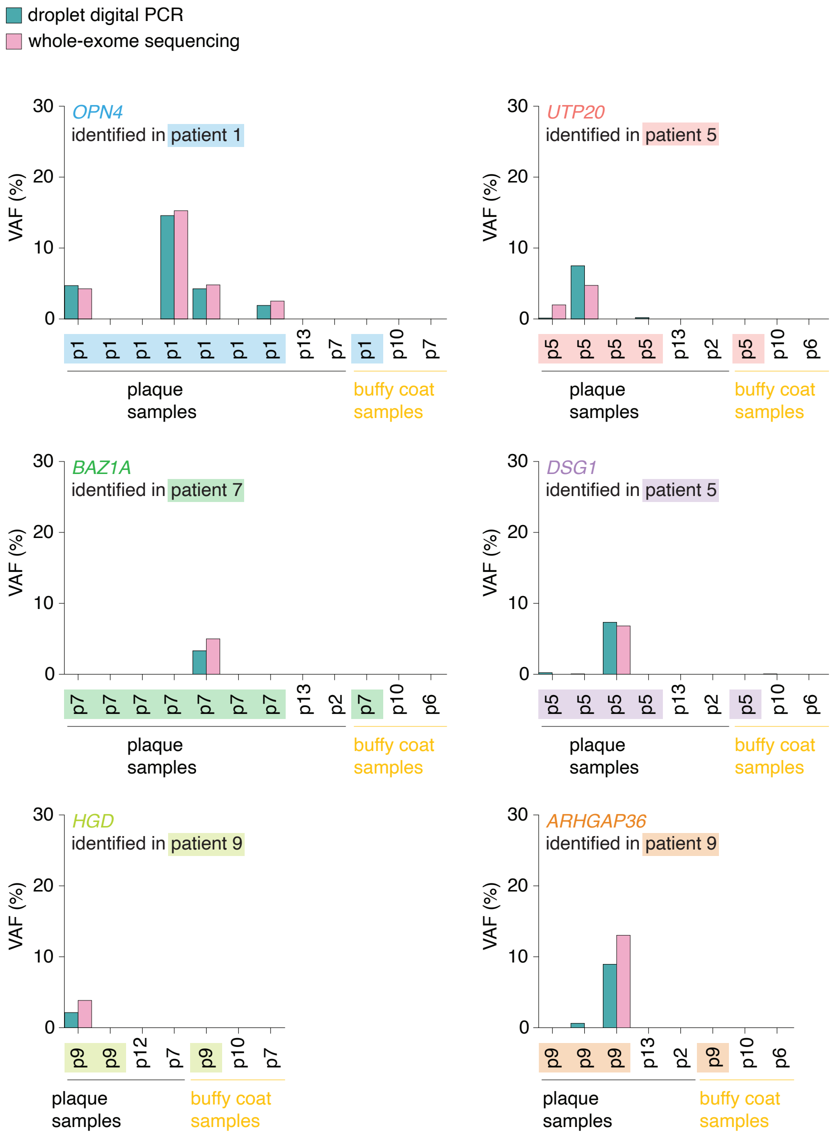

Supplemental Figure S2 I Validation of selected mutations by droplet digital PCR
The plots expand upon the data presented in fig. 1H by incorporating droplet digital PCR data obtained from buffy coats. This additional data serves to validate that the identified mutations were indeed localized to plaque tissue. Furthermore, plaque and buffy coat samples from other patients are included as negative controls in the analysis.

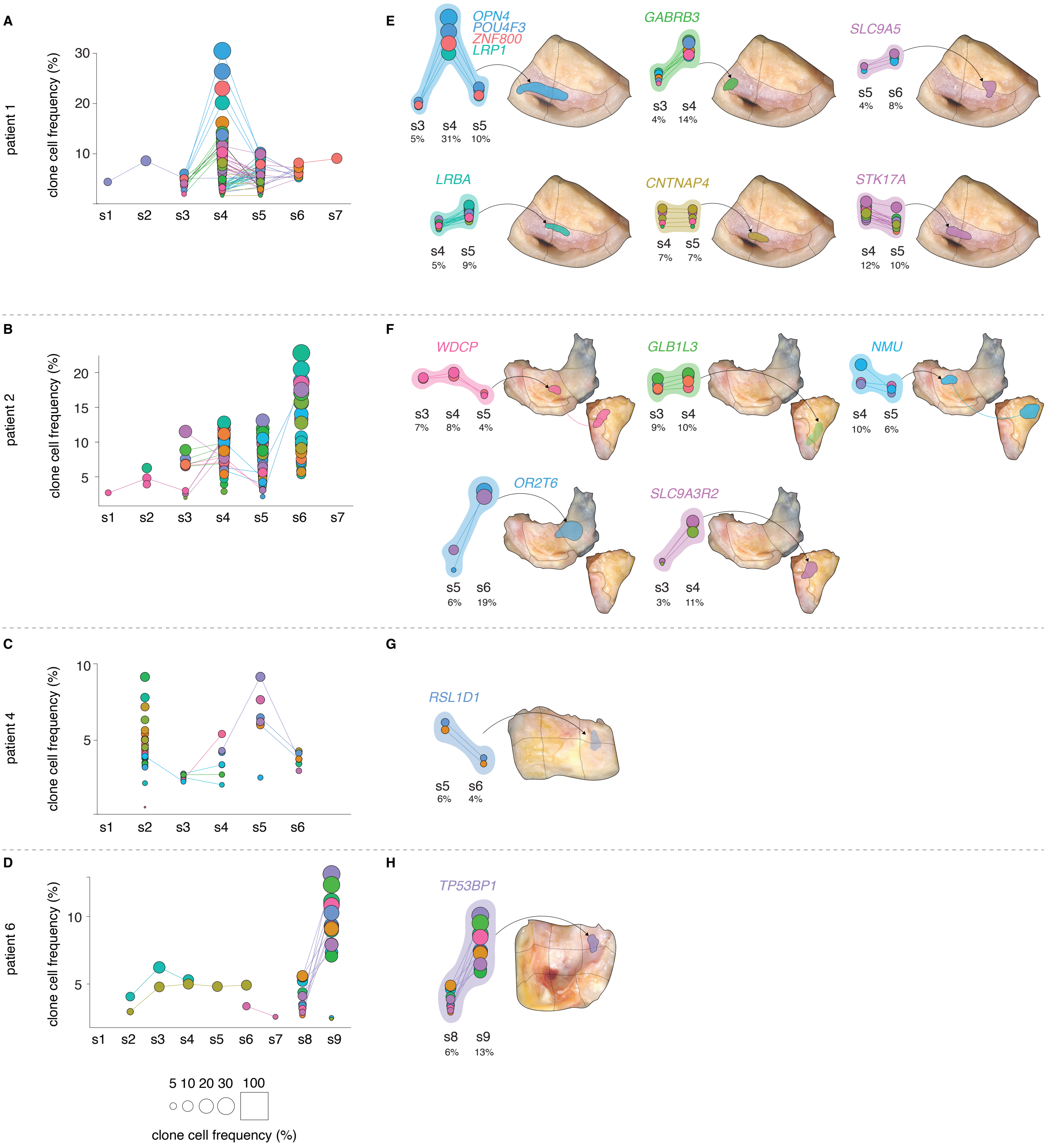

Supplemental Figure S3 I Mutation pattern indicates that some mutations are carried on the same clones
A-D. The plots show the frequency of clone cells (y-axis and dot size) carrying specific somatic mutations in the same plaque samples as presented in fig. 2. Individual mutations are represented by distinct clone cell frequencies, and lines connect mutations shared among multiple samples. Mutations exhibiting consistent sample presence and parallel trends in clone cell frequency are considered to belong to the same clone. By employing this reasoning, distinct mutation sets displaying similar patterns were identified and presumed to originate from the same clone. These sets are visualized in **E-H** wherein clone cell frequencies and gene symbols corresponding to the mutations with highest clone cell frequency is shown.

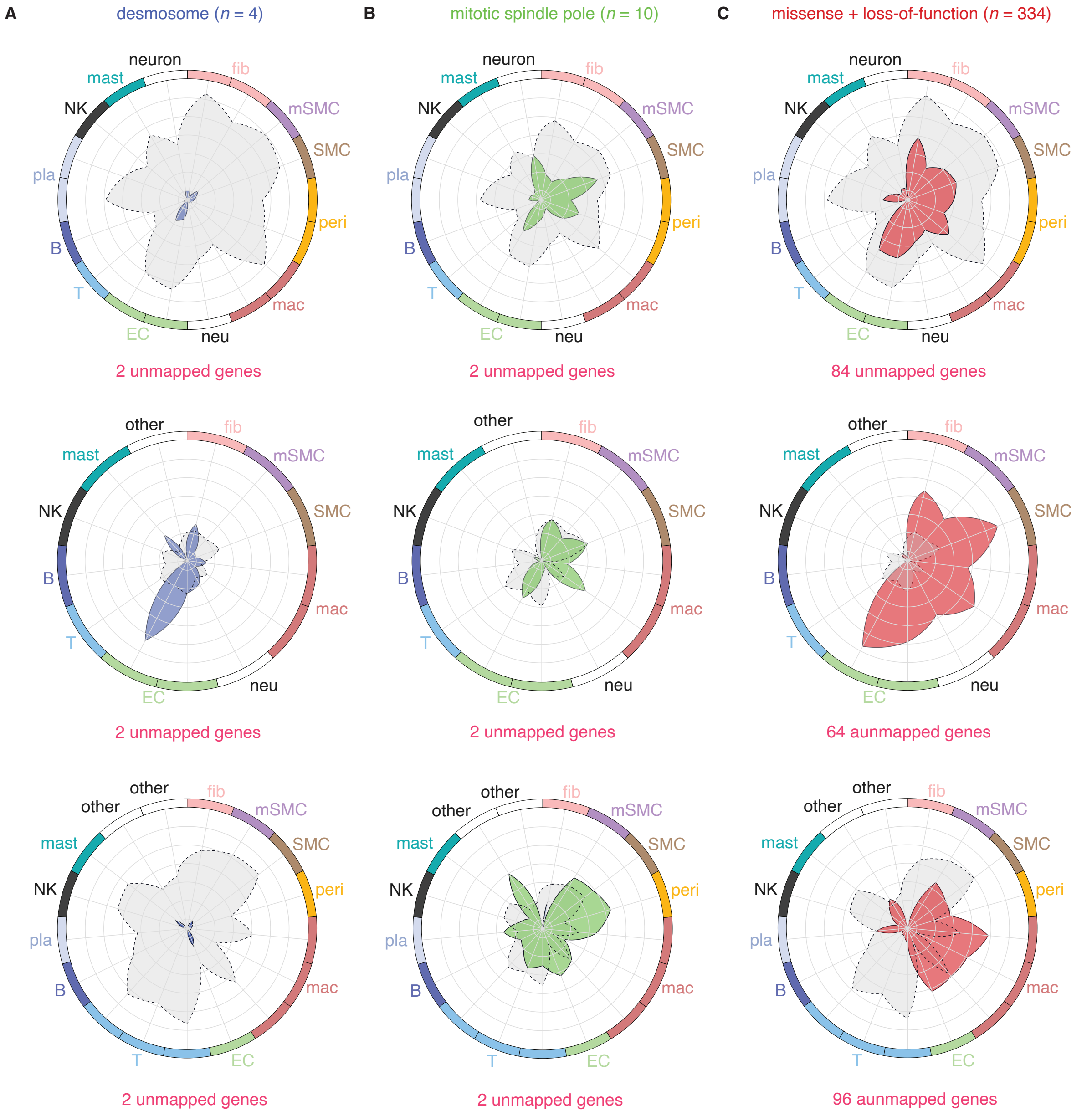

Supplemental Figure S4 | Expression of genes belonging to gene ontology terms "desmosome" and "mitotic spindle pole" in atherosclerosis cell populations.

To evaluate the expression pattern of mutated genes, three single cell RNA sequencing datasets of human plaques were used. The mean expression level of genes belonging to the gene ontology terms desmosome (**A**), mitotic spindle pole (**B**), and all missense and loss-of-function mutations (**C**) in which we found a mutation, is plotted for each cell population. As a reference, the mean expression level of all genes of the atherosclerosis transcriptome was plotted for each dataset in grey for comparison. None of the cell populations showed that mutated genes in the particular category had significantly higher expression than the background gene expression. (Mann-Whitney U test). fib = fibroblast, mSMC = modulated SMC, peri = pericyte, mac = macrophage, neu = neutrophil, EC = endothelial cell, T = T cell, B = B cell, pla = plasma cell, NK = natural killer cell, mast = mast cell, other = un-annotated clusters in original publications.

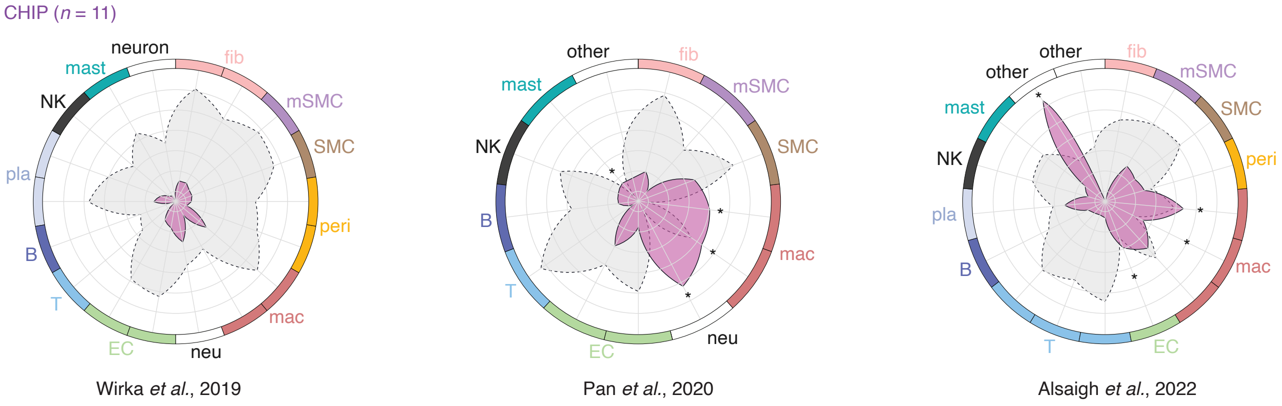

Supplemental Figure S5 I Expression of CHIP-mutated genes identified in the study cohort in atherosclerosis cell populations. To evaluate the expression pattern of mutated CHIP genes identified in the study cohort, three single cell RNA sequencing datasets of human plaques were used. The mean expression level of genes belonging to the CHIP-mutated genes identified in the study cohort is plotted for each cell population. As a reference, the mean expression level of all genes of the atherosclerosis transcriptome was plotted for each dataset in grey for comparison. Asterisks indicate that the identified CHIP-mutated genes have significant higher expression as compared to the background gene population ($p < 0.05$, Mann-Whitney U test). fib = fibroblast, mSMC = modulated SMC, peri = pericyte, mac = macrophage, neu = neutrophil, EC = endothelial cell, T = T cell, B = B cell, pla = plasma cell, NK = natural killer cell, mast = mast cell, other = un-annotated clusters in original publications.
